# Event-Guided Image Reconstruction for Nighttime Dynamic Scenes

**DOI:** 10.3390/jimaging12090399

**Published:** 2026-08-23

**Authors:** Qingjiao Meng, Ji Li, Yan Jin

**Affiliations:** 1 School of Computer Science and Information Security, Guilin University of Electronic Technology, Guilin 541004, China; mjy221206@gmail.com; 2 School of Network Security and Information Technology, Yili Normal University, Yining 835000, China; jy739639988@gmail.com

**Keywords:** nighttime dynamic scenes, nighttime image reconstruction, event camera, low-light imaging, motion deblurring, event–frame fusion

## Abstract

Image reconstruction in nighttime dynamic scenes is challenged by low illumination, long exposure, rapid camera or object motion, and sensor noise. Conventional RGB cameras, therefore, struggle to recover both sufficient brightness and clear structural details in nighttime dynamic scenes. To address this problem, we propose an event-guided image reconstruction method for nighttime dynamic visual perception. The method constructs a multi-channel event voxel representation by jointly encoding event count, event intensity, timestamp distribution, and blurred-frame intensity priors. A parameter-efficient local–global reconstruction network is then designed to restore fine-grained textures and model holistic structures. In addition, edge-alignment and blur-alignment constraints are introduced to improve geometric consistency and imaging plausibility. Experiments on the HQF and REDS datasets show that the proposed method outperforms existing methods in the MSE, PSNR, and SSIM. Compared with DeblurSR, it reduces the MSE by 14.81% on HQF and 10.00% on REDS, while improving the PSNR by 1.603 dB and 1.053 dB, respectively. Qualitative results further show sharper edges, lower structural errors, and better edge consistency. Low illumination, dynamic blur, rapid brightness variation, and event noise are also common degradation factors in nighttime UAV imaging, making the investigated problem technically relevant to that setting. However, because neither REDS nor HQF was acquired during an actual UAV flight, the reported results establish benchmark-level reconstruction performance rather than UAV-specific operational effectiveness.

## 1. Introduction

Image reconstruction in nighttime dynamic scenes is important for visual systems operating under weak illumination and rapid motion. Conventional RGB cameras usually require long exposure to collect sufficient scene irradiance under low-light conditions [1]. However, long exposure introduces motion blur when the camera or objects move during image acquisition [2,3]. The resulting trade-off between imaging brightness and motion sharpness limits reliable recovery of scene structures [4,5]. Although existing low-light image and video enhancement methods can improve brightness and contrast to some extent [6,7,8,9,10], they often struggle to recover critical structures when severe motion blur, degraded edges, and missing local textures are present. RGB-only enhancement may consequently produce detail artifacts, edge distortion, and inconsistent reconstruction in dynamic regions. Therefore, achieving sufficient brightness, clear structures, and motion-consistent reconstruction remains challenging in nighttime dynamic scenes.

Event cameras provide an effective complementary sensing modality for nighttime dynamic visual reconstruction [11,12,13]. As bio-inspired neuromorphic sensors, event cameras asynchronously record pixel-level brightness changes and offer microsecond-level temporal resolution, high dynamic range, and low latency [14]. These properties allow event cameras to capture instantaneous motion cues that are difficult for frame-based cameras to represent under rapid camera or object motion, vibration, and low-light imaging conditions. Event-based blur reconstruction methods can recover not only a single sharp image [15] but also a sequence of latent sharp images corresponding to a blurred frame and its associated event stream [16]. For example, EDI uses high-temporal-resolution brightness changes provided by events to constrain the blur-formation process, thereby recovering latent sharp images from a blurred observation [17]. Such methods offer strong physical interpretability, but their fixed temporal integration strategy is insufficient for handling complex blur caused by varying motion speeds, motion directions, and camera shake, which limits their performance in highly dynamic scenes.

In recent years, learning-based event image reconstruction methods have further improved reconstruction performance. E2VID reconstructs images from events using a recurrent convolutional neural network and incrementally updates previous reconstructions through a recurrent U-Net architecture, but its reliance on short-term historical information may limit reconstruction accuracy [18]. FireNet improves inference efficiency by reusing historical features with a lightweight recurrent structure, although its reconstruction quality remains limited [19]. eSL-Net exploits events to compensate for high-frequency and motion cues while introducing sparse constraints to suppress noise amplification; nevertheless, severe blur and noise under long exposure may prevent events from accurately describing complete motion trajectories, resulting in insufficient detail recovery [20]. EventSR improves reconstruction accuracy through a multi-stage pipeline involving low-quality image reconstruction, enhancement, upsampling, and adversarial learning, but its complex workflow still shows limited adaptability to nighttime dynamic scenes [21]. E-CIR performs joint deblurring and interpolation in the continuous-time domain using event-based regularization, whereas threshold drift and event noise may weaken brightness recovery and cause high-frequency detail loss [22]. Recent DeblurSR models blurred frames as event-driven time-to-intensity mappings and use spiking representations for efficient temporal encoding. However, its performance relies heavily on accurate event capture and predefined motion assumptions, making it less robust to irregular motion and complex nighttime scene variations [23]. Overall, existing methods still face three major challenges in nighttime dynamic scenes: low illumination and motion blur jointly degrade image structures; event noise and incomplete motion responses weaken dynamic-region recovery; and many existing networks lack joint constraints on local textures, global structures, and physical imaging consistency.

To address these challenges, this paper proposes an event–frame fusion image reconstruction method for nighttime dynamic scenes. The proposed method establishes a complementary mechanism between event cameras and frame-based cameras by jointly exploiting high-temporal-resolution motion cues from event streams and spatial texture information from intensity frames, thereby enhancing both dynamic-region representation and static structure recovery. Specifically, we first perform complementary event–frame fusion to obtain joint features containing motion-indicative cues and spatial textures. Then, we design a three-stage local–global–fusion reconstruction architecture. The local branch restores fine-grained textures, the global branch models holistic scene structures, and the fusion module adaptively integrates local details and global semantics through attention mechanisms. Furthermore, we introduce a dual-supervision strategy consisting of an edge-alignment constraint and a blur-alignment constraint. The edge-alignment constraint preserves contour structures and geometric boundaries, while the blur-alignment constraint enforces imaging consistency between the reconstructed sharp image and the original blurred observation based on the physical blur-formation process. With these designs, the proposed method can better adapt to different types of blur caused by varying speeds, motion directions, and camera vibration, while maintaining image sharpness, structural fidelity, and physical plausibility. Low illumination, dynamic blur, rapid brightness variation, and event noise are also frequently encountered in nighttime UAV imaging. This correspondence makes the investigated degradation model technically relevant to UAV imagery, but it does not substitute for evaluation using data acquired during actual UAV flights.

The individual operations used in the framework, including voxelization, depthwise separable convolution, channel and spatial attention, and Swish activation, are established techniques and are not claimed as independent methodological innovations. The technical contribution lies in their task-specific coordination with a multi-statistic event–frame representation and differentiable event-guided structural and imaging constraints. This coordinated design addresses the simultaneous occurrence of weak frame intensity, motion blur, sparse or noisy event responses, and structural degradation in nighttime dynamic reconstruction.

The main highlights of this paper are summarized as follows:

1. This paper proposes an event–frame fusion image reconstruction method for nighttime dynamic scenes. It effectively alleviates image degradation caused by low illumination, long exposure, platform vibration, and high-speed motion.
2. A multi-statistic event–frame representation is constructed by encoding event count, average polarity response, and normalized timestamp within 13 temporal bins and incorporating normalized blurred-frame intensity before feature extraction. The resulting 39-channel representation preserves complementary spatial and temporal information under low illumination and unreliable event responses.
3. A representation-aware local–global reconstruction architecture coordinates local texture recovery, global structural modeling, and channel–spatial feature selection. Depthwise separable convolution, attention operations, and Swish activation are employed as established building blocks within this coordinated pathway rather than claimed as individual innovations.
4. A differentiable event-guided dual-alignment objective combines reliable event-boundary alignment with an event-guided reblur operator. It constrains the single-frame reconstruction from geometric and exposure-formation perspectives.
5. Experimental results on the HQF and REDS datasets demonstrate that the proposed method outperforms existing mainstream approaches while reducing resource requirements, showing strong robustness and generalization capability on the evaluated benchmarks. Because these datasets were not acquired during UAV flights, UAV-specific effectiveness is not claimed.

## 2. Related Work

### 2.1. Event-Based Image Reconstruction

Conventional frame-based cameras generate images by integrating photocurrent over the exposure time, providing rich spatial textures and absolute intensity information. However, under nighttime low-light conditions and rapid motion, frame images are easily degraded by noise, underexposure, and motion blur [23]. In contrast, event cameras asynchronously record pixel-level brightness changes and generate events only when the brightness variation exceeds a predefined threshold. Therefore, they offer high temporal resolution, high dynamic range, and low latency. Nevertheless, event streams mainly describe brightness changes rather than complete grayscale or color information, and they may become incomplete in weak-texture, low-light, or noisy scenes.

### 2.2. Event–Frame Fusion Methods

Event–frame fusion has therefore become an important direction in event-based visual reconstruction. Frame images provide stable spatial structures and texture information, while event streams complement them with temporal variations and edge responses in fast-moving regions. By aligning and fusing two-dimensional image information with three-dimensional event streams, existing methods can jointly exploit the spatial representation ability of frame cameras and the dynamic sensing ability of event cameras. This fusion strategy has shown advantages in image reconstruction, deblurring, low-light enhancement, and high-dynamic-range scene recovery. Compared with methods relying only on frames or events, event–frame fusion can better alleviate insufficient brightness, motion blur, and detail loss in nighttime dynamic scenes. These degradation factors are also encountered in nighttime UAV imaging, but application-level performance depends on additional platform-specific factors and must be evaluated using real flight data. Challenges remain due to spatiotemporal resolution inconsistency, event noise, low-light degradation, and complex motion blur, which motivate further method design in subsequent sections.

## 3. Proposed Method

Given a blurred frame and its associated event stream acquired during the exposure interval, the proposed method reconstructs a single latent sharp image through event representation, event–frame fusion, local–global feature reconstruction, and dual-constraint optimization. We first introduce the event-generation model and define the event representation used in this work, followed by the detailed network architecture and objective functions.

### 3.1. Event-Generation Model

Each event is denoted by $e_{i}=\left(x_{i},y_{i},t_{i},p_{i}\right)$, where $\left(x_{i},y_{i}\right)$ are the pixel coordinates, $t_{i}$ is the event timestamp, and the polarity $p_{i}\in \left\{-1,+1\right\}$ is a binary constant indicating an increase or decrease in brightness. The event stream is written as E(x,y,t). The grayscale frame image is denoted by $I_{f}\left(x,y\right)$. By fusing E(x,y,t) and $I_{f}\left(x,y\right)$, we aim to reconstruct the grayscale image.

**Figure 1:**
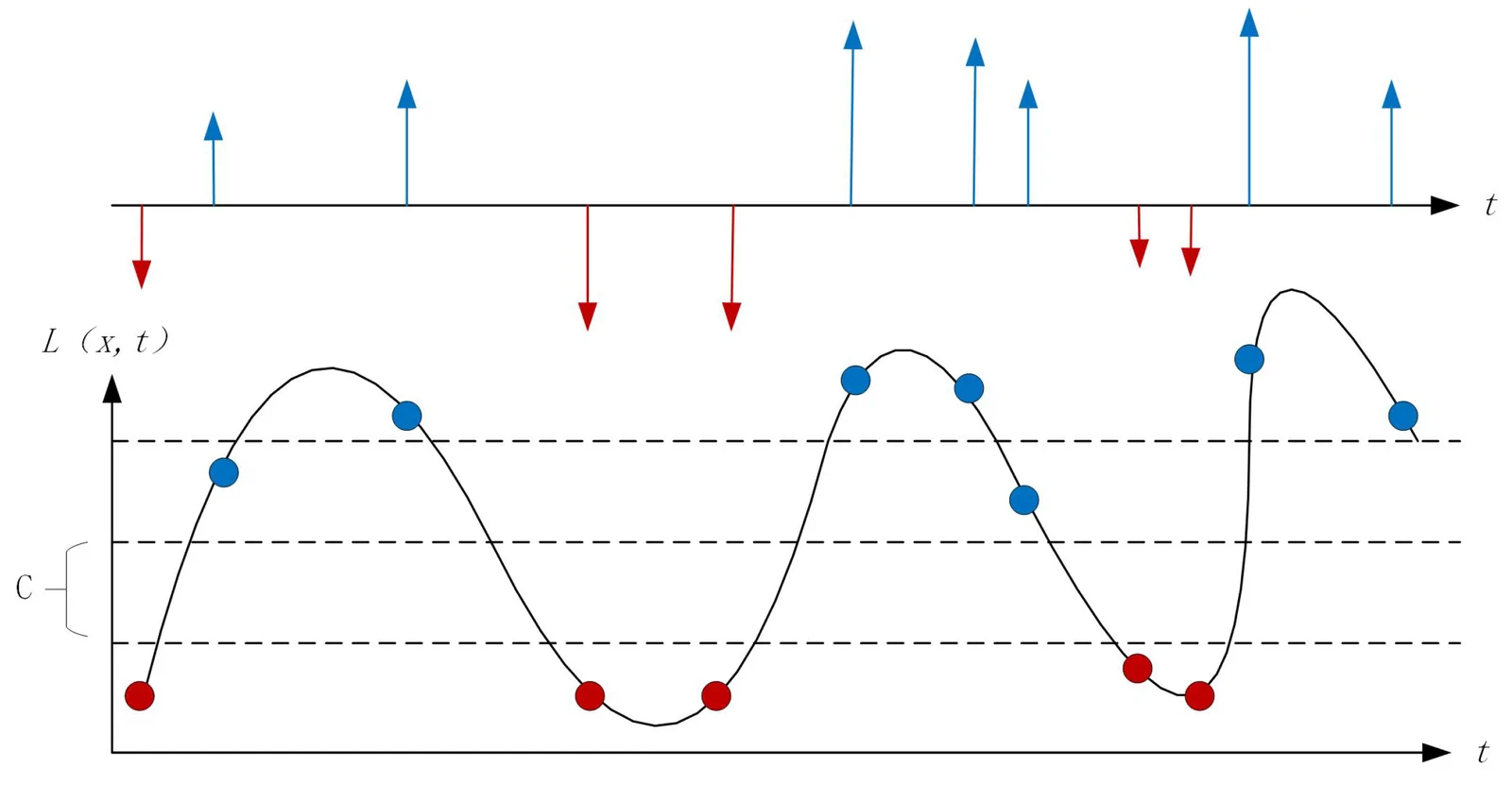
Event signal generation model. Blue markers and upward arrows denote positive-polarity events, whereas red markers and downward arrows denote negative-polarity events. The black curve represents the log-intensity signal.

The event-generation mechanism is illustrated in Figure 1. Let L(x,y,t)=logI(x,y,t) denote logarithmic intensity and define the temporal log-intensity change as ΔL(x,y,t)=L(x,y,t)−L(x,y,t−Δt). The positive quantities $C_{+}$ and $C_{-}$ denote the magnitudes of the positive and negative contrast thresholds, respectively. A positive event is generated when ΔL reaches $C_{+}$, whereas a negative event is generated when ΔL reaches $-C_{-}$. Figure 1 illustrates the symmetric special case $C_{+}=C_{-}=C$.

(1) $$\begin{array}{c} p\left(x,y,t\right)=\left\{\begin{array}{cc} +1, & \Delta L\left(x,y,t\right)\geq C_{+}\\ -1, & \Delta L\left(x,y,t\right)\leq -C_{-} \end{array}\right. \end{array}$$

No event is generated when $-C_{-}<\Delta L\left(x,y,t\right)<C_{+}$. The two threshold magnitudes need not be identical for a physical event camera because they depend on sensor bias settings and may exhibit pixel-level variability. The polarity of every triggered event nevertheless remains $p_{i}\in \left\{-1,+1\right\}$.

### 3.2. Event Voxel Representation

As discussed in the previous section, event cameras provide high-temporal-resolution motion responses. However, raw event streams are usually sparse, asynchronous, and sensitive to noise, making them unsuitable for direct input into convolutional neural networks. In addition, using only event counts or timestamp statistics is insufficient to fully describe local motion intensity, temporal variation, and spatial structure in nighttime dynamic scenes. Under low illumination, long exposure, and rapid motion, event distributions may suffer from local missing responses or noise disturbance, which makes it difficult for reconstruction networks to accurately identify blurred regions and motion variations. Therefore, it is necessary to convert raw event streams into a structured representation while preserving spatial distribution, temporal response, and motion intensity information. To this end, we convert raw event data into a fixed-size multi-channel voxel grid. Let the event stream be denoted as $E=e_{i}{i=1}^{N}$, where each event is represented as $e_{i}=\left(x_{i},y_{i},t_{i},p_{i}\right)$. Here, $x_{i}$ and $y_{i}$ denote the spatial coordinates, $t_{i}$ denotes the timestamp, and $p_{i}$ represents the event polarity or event intensity. Since image reconstruction requires spatial alignment with the input frame, the event stream is divided into a spatial grid with the same resolution as the image. Events within a selected temporal window $\left[t_{0},t_{1}\right]$ are normalized and grouped to obtain an event voxel grid of size $H\times W\times \begin{array}{c} D \end{array}$, where $\begin{array}{c} D \end{array}$ denotes the number of voxel feature channels. Following the temporal partition used in DeblurSR [23], the exposure interval $\left[t_{0},t_{1}\right]$ is uniformly divided into B=13 non-overlapping temporal bins. The bins are defined as

(2) $$\begin{array}{c} T_{b}=\left\{\begin{array}{cc} \left[t_{0}+\left(b-1\right)\Delta t,\;t_{0}+b\Delta t\right), & b=1,\ldots ,B-1,\\ \left[t_{0}+\left(B-1\right)\Delta t,\;t_{1}\right], & b=B, \end{array}\right.\;\Delta t=\frac{t_{1}-t_{0}}{B} \end{array}.$$

The first B−1 bins are left-closed and right-open, whereas the last bin is closed at $t_{1}$. Therefore, every event in the exposure interval, including an event with timestamp $t_{i}=t_{1}$, is assigned to exactly one temporal bin. Rather than using only polarity-separated event counts, the proposed representation retains three complementary statistics in every temporal bin: event count, average polarity response, and average normalized timestamp. Consequently, the voxel depth is D=3B=39. Specifically, three basic event channels are constructed for each spatial location and temporal bin: event count, event intensity, and average timestamp. The event-count channel describes the density of event responses and reflects the activity level of local motion or brightness changes. The event intensity channel aggregates polarity or intensity responses within each voxel, representing the direction and magnitude of local brightness variations. The average timestamp channel records the temporal distribution of events within the voxel and reflects the temporal consistency of local motion. The three event features are calculated as follows:

(3) $$\begin{array}{c} \left\{\begin{array}{cc} E_{b}\left(x,y\right) & =\sum_{i=1}^{N}1\left[x_{i}=x,\;y_{i}=y,\;t_{i}\in T_{b}\right],\\ I_{b}\left(x,y\right) & =\left\{\begin{array}{cc} \frac{\sum_{i=1}^{N}p_{i}1\left[x_{i}=x,\;y_{i}=y,\;t_{i}\in T_{b}\right]}{E_{b}\left(x,y\right)}, & E_{b}\left(x,y\right)>0,\\ 0, & E_{b}\left(x,y\right)=0, \end{array}\right.\\ T_{b}\left(x,y\right) & =\left\{\begin{array}{cc} \frac{\sum_{i=1}^{N}{\tilde{t}}_{i}1\left[x_{i}=x,\;y_{i}=y,\;t_{i}\in T_{b}\right]}{E_{b}\left(x,y\right)}, & E_{b}\left(x,y\right)>0,\\ 0, & E_{b}\left(x,y\right)=0, \end{array}\right.. \end{array}\right. \end{array}$$

Here, ${\tilde{t}}_{i}=\left(t_{i}-t_{0}\right)/\left(t_{1}-t_{0}\right)$ is the normalized timestamp and 1[·] is the bin-membership indicator. When $E_{b}\left(x,y\right)>0$, $I_{b}\left(x,y\right)$ and $T_{b}\left(x,y\right)$ denote the average polarity response and average normalized timestamp, respectively. When no event falls into a spatial–temporal bin, all three statistics are set to zero. This conditional definition prevents division by zero and ensures that an empty bin introduces no artificial event response. A binary validity mask is retained in the implementation to distinguish an empty bin from a non-empty bin whose signed polarity responses cancel to zero. A larger average timestamp indicates that events are more concentrated in the later part of the temporal window, whereas a more dispersed timestamp distribution may indicate continuous motion, complex dynamic changes, or noise disturbance. By jointly encoding event count, event intensity, and timestamp distribution, the constructed voxel grid provides a more comprehensive description of spatiotemporal variations in nighttime dynamic scenes than single event statistics, offering more stable input features for subsequent event–frame fusion and image reconstruction. Given an input blurred image $I_{\mathrm{blur}}$ and its corresponding event stream E acquired during the exposure interval, the proposed network reconstructs a single latent sharp image,

(4) $$\begin{array}{c} \hat{L}=F_{\theta }\left(I_{\mathrm{blur}},E\right) \end{array},$$

where θ denotes the trainable network parameters. The symbol B=13 denotes the number of temporal bins used to discretize the event stream, b∈{1,…,B} is the temporal-bin index, and $T_{b}$ denotes the interval of the *b*th bin. Accordingly, *B* is used exclusively for the temporal-bin number, whereas $I_{\mathrm{blur}}$ denotes the input blurred image.

### 3.3. Fusion with Blurred Frames

To further enhance the spatial intensity information in the event voxel representation, we introduce the intensity variation in the blurred frame into the event voxel grid. Since event streams record pixel-level brightness changes in a three-dimensional spatiotemporal form, while nighttime blurred frames mainly provide two-dimensional spatial intensity observations, there exists an inherent difference in data format and representation dimension. Therefore, before event–frame fusion, spatial consistency between the blurred frame and the event voxel grid should be ensured.

**Figure 2:**
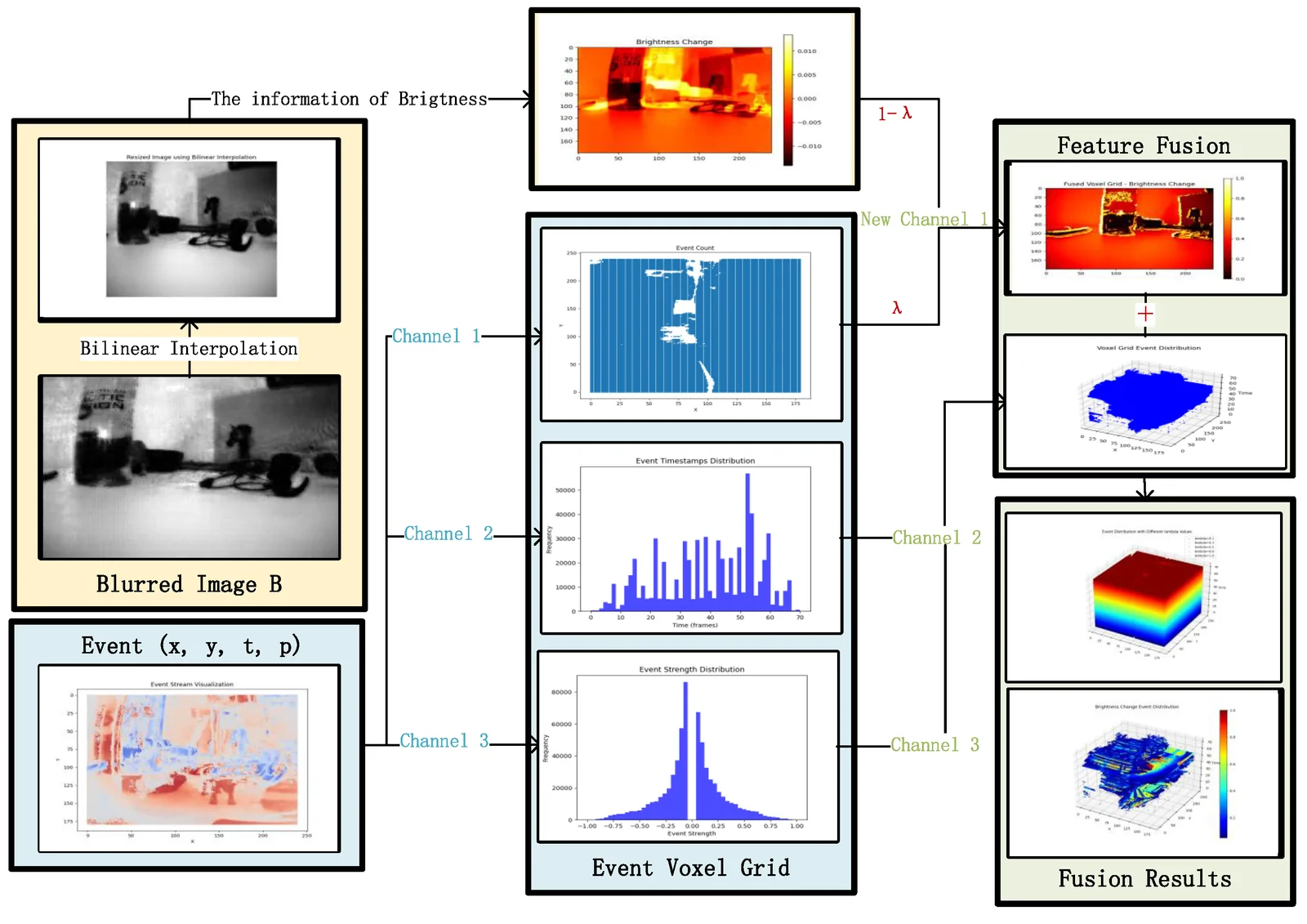
Illustration of the proposed event–frame complementary voxel representation for nighttime dynamic visual reconstruction. The blurred frame is first processed by bilinear interpolation to extract brightness variation information, while the asynchronous event stream is encoded into event-count, timestamp distribution, and event intensity channels. These complementary channels are then fused to construct a multi-channel voxel representation that integrates spatial texture cues from the frame camera and high-temporal-resolution motion cues from the event camera. This representation provides robust spatiotemporal features for low-light dynamic reconstruction.

As shown in Figure 2, bilinear interpolation is adopted to resample the blurred frame so that its spatial size is consistent with that of the event voxel grid. Bilinear interpolation can better preserve local intensity distribution and spatial continuity during scale transformation, avoiding block artifacts and abrupt detail changes caused by nearest-neighbor interpolation. After resampling, each pixel location in the blurred frame can be aligned with the corresponding spatial unit in the event voxel grid, ensuring that frame-based intensity information is accurately mapped into the event representation.

Before fusion, the two input modalities are normalized to comparable numerical ranges. For an 8-bit blurred frame $I_{\mathrm{blur}}$, fixed-range normalization is used as ${\tilde{I}}_{\mathrm{blur}}\left(x,y\right)=I_{\mathrm{blur}}\left(x,y\right)/255$, yielding ${\tilde{I}}_{\mathrm{blur}}\in \left[0,1\right]$ while preserving the relative brightness relationships among different images. After spatial resampling, the resulting normalized frame intensity is denoted by ${\tilde{I}}_{f}$. For the non-negative event-count response *E*, we first apply logarithmic compression, $E^{\log}=\log\left(1+E\right)$, and then perform percentile-based normalization:

(5) $$\begin{array}{c} \tilde{E}=\mathrm{clip}\left(\frac{E^{\log}}{q_{0.99}+\epsilon },0,1\right) \end{array},$$

where $q_{0.99}$ is the 99th percentile of the non-zero values in $E^{\log}$ and $\epsilon =10^{-6}$ prevents division by zero. This operation reduces the influence of a small number of unusually large event counts while retaining relative event-response differences. Event timestamps are normalized within each accumulation window as ${\tilde{t}}_{i}=\left(t_{i}-t_{0}\right)/\left(t_{1}-t_{0}\right)$, whereas polarity remains signed in its separate channel. Thus, the frame intensity and event-count channels entering the weighted fusion are both bounded to [0,1]. If the complete event-count tensor contains no non-zero value in an accumulation window, $\tilde{E}$ is directly set to zero; otherwise, $q_{0.99}$ is calculated only from its non-zero responses.

In nighttime dynamic scenes, although blurred frames are degraded by low illumination and motion blur, they still preserve global intensity distribution and partial static structure information. In contrast, event streams provide high-temporal-resolution responses in fast-moving regions. Based on this complementarity, the resampled blurred-frame intensity information is fused with the event-count channel in the event voxel grid. The event-count channel reflects the activity level of local brightness changes, while the blurred-frame intensity information supplements the missing spatial intensity prior of the event stream. Their combination enhances spatial intensity representation while preserving temporal event responses, thereby providing more stable input features for subsequent nighttime dynamic image reconstruction. The fusion process is formulated in Equation (6):

(6) $$\begin{array}{c} E_{fusion}\left(x,y,t\right)=\lambda_{f}{\tilde{I}}_{f}\left(x,y\right)+\left(1-\lambda_{f}\right)\tilde{E}\left(x,y,t\right) \end{array},$$

where $E_{\mathrm{fusion}}\left(x,y,t\right)$ denotes the fused event–frame voxel response rather than a simple event-count statistic. $\lambda_{f}$ is the event–frame fusion weight that controls the relative contribution of normalized blurred-frame intensity and normalized event-count information. A smaller $\lambda_{f}$ makes the fused representation rely more on the high-temporal-resolution motion responses from event streams, whereas a larger $\lambda_{f}$ emphasizes the spatial intensity distribution of the blurred frame. In all experiments, $\lambda_{f}$ is a fixed global scalar shared across all spatial locations, temporal bins, and input samples. It is not learned by the network and does not vary spatially or temporally. Its value is selected once on the REDS validation split through the parameter-sensitivity experiment described in Section 4.4.1. After selection, $\lambda_{f}$ is fixed at 0.5 for all subsequent experiments, including the evaluation on HQF, without any additional tuning. For an empty event bin, ${\tilde{E}}_{b}\left(x,y\right)=0$ before fusion. The corresponding fused response therefore reduces to $\lambda_{f}{\tilde{I}}_{f}\left(x,y\right)$. Thus, an empty bin produces no false motion response, while the aligned blurred frame still supplies a spatial intensity prior in weak-response regions.

Specifically, $I_{f}\left(x,y\right)$ denotes the resampled intensity information of the blurred frame, which provides global intensity distribution and spatial structural priors in nighttime scenes. The event voxel grid records event count, event intensity, and timestamp distribution within local regions, reflecting temporal characteristics of pixel-level brightness changes. These two modalities are complementary: frame images provide stable spatial textures and intensity priors, while event streams provide high-temporal-resolution motion cues. Through weighted fusion, the proposed representation introduces spatial intensity information from the frame image while preserving event-based dynamic responses, thereby enhancing the spatiotemporal contextual representation of the voxel grid. This fusion mechanism helps alleviate low-light degradation, motion blur, and locally missing event responses in nighttime dynamic scenes, providing more stable input features for subsequent image reconstruction.

### 3.4. Dual-Branch Feature Fusion Network

Through the above event–frame fusion process, we obtain a joint feature representation that incorporates the intensity prior of the blurred frame, event spatial locations, event counts, event intensities, and timestamp distributions. This representation preserves both the spatial texture information of frame images and the high-temporal-resolution motion responses of event streams, thereby providing a more stable spatiotemporal input for image reconstruction in nighttime dynamic scenes. The fused features are then fed into the proposed local–global dual-branch feature fusion network to further enhance dynamic-region details and holistic structural representations.

**Figure 3:**
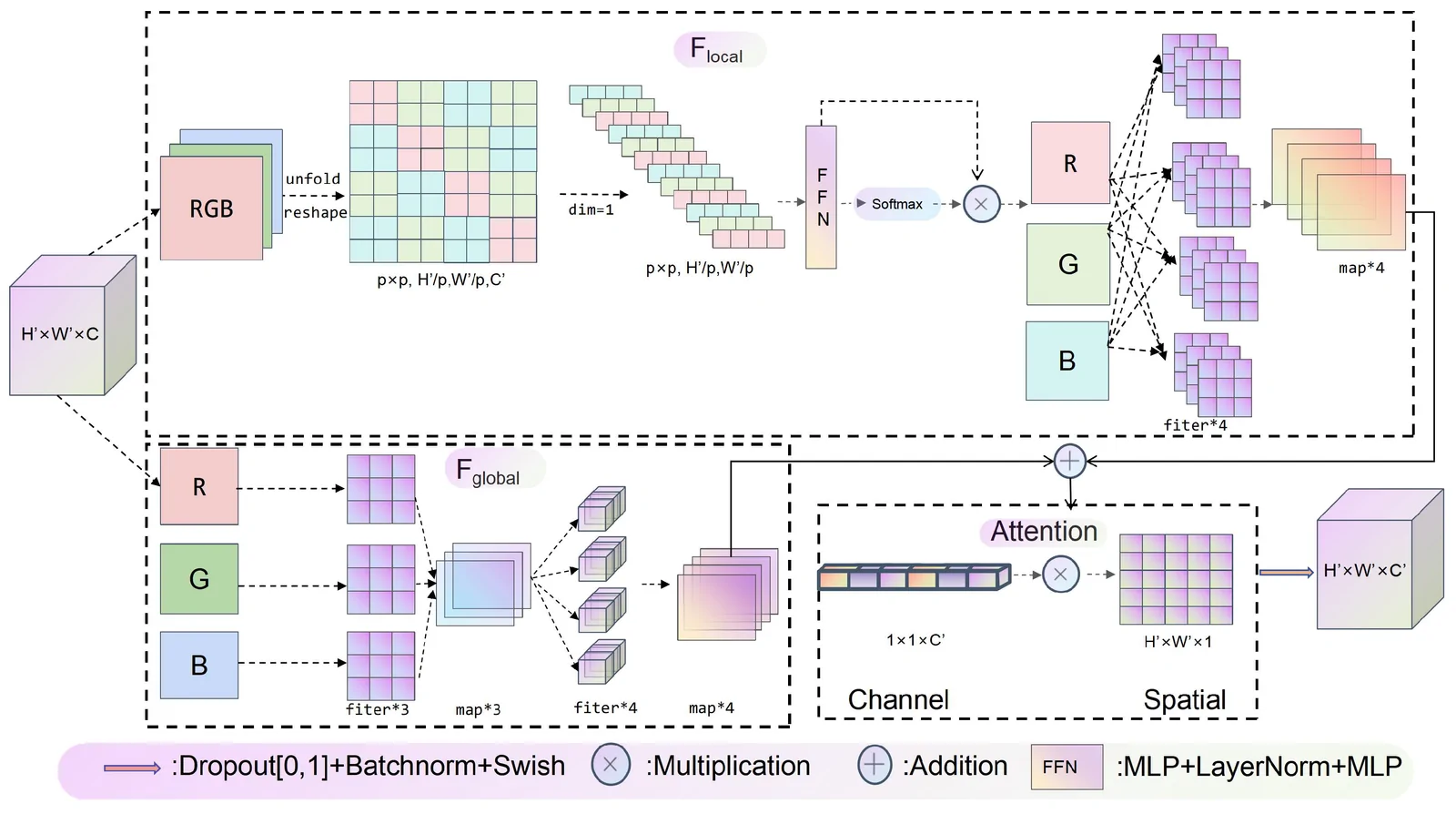
Local–global dual-branch feature fusion network for nighttime dynamic reconstruction. The image intensity information is first weighted and fused with the event voxel grid containing spatial location, event count, event intensity, and timestamp distribution to obtain joint event–frame features. The fused features are then fed into a local–global dual-branch network, where the local branch extracts fine-grained textures and edge structures, while the global branch models holistic scene structures and long-range dependencies. Channel and spatial attention mechanisms are further introduced in the fusion stage to emphasize informative feature channels and focus on dynamically blurred regions. The output feature map enhances both local-detail recovery and global structural consistency, providing a stable representation for nighttime dynamic image reconstruction.

As shown in Figure 3, let the input feature be denoted as $F\in R^{H^{\prime }\times W^{\prime }\times C^{\prime }}$. The network first applies convolutional operations to perform an initial feature mapping, producing an enhanced feature representation $F^{\prime }\in R^{H^{\prime }\times W^{\prime }\times C^{\prime }}$. Subsequently, feature modeling is conducted through two complementary branches, namely, a local branch and a global branch. The local branch extracts fine-grained textures and edge information through local window partitioning, feature unfolding, and convolutional projection, with particular emphasis on enhancing local structural details in blurred regions under nighttime dynamic conditions. In contrast, the global branch captures holistic scene layouts and large-scale motion variations by aggregating global features and modeling long-range dependencies with multilayer perceptrons. Since nighttime dynamic reconstruction requires both local texture restoration and global structural consistency, relying on a single-scale or single-path representation is insufficient to achieve stable reconstruction.

To further improve feature selection, channel attention and spatial attention mechanisms are introduced in the fusion stage. Channel attention emphasizes informative feature channels related to intensity variation, motion response, and structural recovery, whereas spatial attention focuses on regions with severe blur or significant motion changes. Finally, features extracted by the local and global branches are adaptively fused to obtain the fused representation $F_{lg}\in R^{H^{\prime }\times W^{\prime }\times C^{\prime }}$, which is then used as the input to the subsequent image reconstruction module. This local–global collaborative modeling strategy improves representation of nighttime dynamic scenes through a parameter-efficient architecture.

#### 3.4.1. Local Branch Strategy

The local branch is designed to enhance local textures, edge structures, and fine-grained motion responses in the fused feature representation. In nighttime dynamic scenes, low illumination, motion blur, and event noise often degrade local structural details. Therefore, it is necessary to explicitly model neighboring spatial features within local regions.

As shown in Figure 3, given the input feature $F^{\prime }\in R^{H^{\prime }\times W^{\prime }\times C^{\prime }}$, we first apply unfold and reshape operations to partition it into local windows. Let the window size be p×p. The feature is then reorganized into $R^{p\times p\times \frac{H^{\prime }}{p}\times \frac{W^{\prime }}{p}\times C^{\prime }}$, where *p* denotes the spatial scale of each local window. This operation explicitly unfolds continuous local regions without destroying spatial neighborhood relationships, facilitating subsequent modeling of local textures and edge variations.

Then, channel-wise average pooling is applied to the unfolded local features to suppress redundant responses and highlight the dominant structural information within each local region. To further improve the nonlinear representation capability of local features, a Feed-Forward Network (FFN) is introduced to map the pooled features. Given an input vector $\tilde{x}$, the output $\tilde{y}$ of the FFN is formulated as:

(7) $$\tilde{y}=\mathrm{FFN}\left(\tilde{x}\right)={\mathrm{Linear}}_{2}\left(\mathrm{ReLU}\left({\mathrm{Linear}}_{1}\left(\tilde{x}\right)\right)\right).$$

The linear transformations are defined as:

(8) $${\mathrm{Linear}}_{1}\left(\tilde{x}\right)=W_{1}\tilde{x}+b_{1},\;{\mathrm{Linear}}_{2}\left(\tilde{x}\right)=W_{2}\tilde{x}+b_{2},$$

where $W_{1}\in R^{d_{\mathrm{low}}\times d_{\mathrm{high}}}$, $W_{2}\in R^{d_{\mathrm{high}}\times d_{\mathrm{low}}}$, $b_{1}\in R^{d_{\mathrm{high}}}$, and $b_{2}\in R^{d_{\mathrm{low}}}$. Here, $d_{\mathrm{low}}$ denotes the input feature dimension, while $d_{\mathrm{high}}$ denotes the hidden dimension of the FFN. Through this expansion-and-projection process, the FFN captures more complex local patterns in a higher-dimensional space and preserves the most discriminative information after dimensionality reduction.

Finally, the processed local features are fed into a 3×3 convolutional layer for spatial aggregation, producing the local branch output $F_{\mathrm{local}}\in R^{H^{\prime }\times W^{\prime }\times C^{\prime }}$. This local branch effectively enhances edge contours, local textures, and fine-grained motion variations in nighttime dynamic scenes, providing a more reliable local structural representation for subsequent local–global feature fusion.

#### 3.4.2. Global Branch Strategy

The global branch is designed to model holistic structural distributions and large-scale motion variations in nighttime dynamic scenes. Unlike the local branch, which focuses on fine-grained textures and edge details, the global branch aims to capture scene layout, dynamic-region correlations, and contextual dependencies among different spatial locations. In nighttime low-light and motion-blurred scenes, relying only on local features may lead to discontinuous structural recovery or inconsistent reconstruction in dynamic regions. Therefore, we design a lightweight global branch to enhance global structural representation while controlling computational cost.

As shown in Figure 3, given the input feature $F^{\prime }\in R^{H^{\prime }\times W^{\prime }\times C^{\prime }}$, the global branch first adopts depthwise separable convolution for feature modeling. Specifically, depthwise convolution is applied to each channel independently to extract spatial structural responses within individual channels. Then, a 1×1 pointwise convolution is used to fuse cross-channel information, producing a feature representation with enhanced global contextual capability. Compared with standard convolution, depthwise separable convolution reduces the parameter and computational cost of this branch, making it suitable for efficient dynamic reconstruction.

Let the convolution kernel size be $D_{k}\times D_{k}$, the number of input channels be *M*, the number of output channels be *N*, and the spatial size of the feature map be $D_{F}\times D_{F}$. The number of parameters of depthwise convolution is $D_{k}\times D_{k}\times M$, and its computational cost is:

(9) $$D_{k}\times D_{k}\times M\times D_{F}\times D_{F}.$$

The subsequent 1×1 pointwise convolution fuses information across channels, with M×N parameters and a computational cost of:

(10) $$M\times N\times D_{F}\times D_{F}.$$

Therefore, the total computational cost of depthwise separable convolution in the global branch is:

(11) $$D_{k}\times D_{k}\times M\times D_{F}\times D_{F}+M\times N\times D_{F}\times D_{F}.$$

In contrast, the computational cost of standard convolution is:

(12) $$D_{k}\times D_{k}\times M\times N\times D_{F}\times D_{F}.$$

The ratio between the computational cost of depthwise separable convolution and standard convolution is given by:

(13) $$\frac{D_{k}\times D_{k}\times M\times D_{F}\times D_{F}+M\times N\times D_{F}\times D_{F}}{D_{k}\times D_{k}\times M\times N\times D_{F}\times D_{F}}=\frac{1}{N}+\frac{1}{D_{k}^{2}}.$$

When $D_{k}=3$, the ratio becomes $\frac{1}{N}+\frac{1}{9}$. As the number of output channels *N* increases, the computational cost gradually approaches approximately $\frac{1}{9}$ of that of standard convolution, indicating that the proposed structure can achieve effective global feature modeling with substantially lower computational overhead.

Finally, the global branch outputs $F_{\mathrm{global}}\in R^{H^{\prime }\times W^{\prime }\times C^{\prime }}$, which represents holistic scene structures, long-range contextual relationships, and large-scale dynamic variations. This branch enhances the global representation capability of the network for nighttime dynamic scenes while maintaining low computational complexity, providing stable structural priors for subsequent local–global feature fusion.

#### 3.4.3. Feature Fusion Method

After feature extraction by the local and global branches, we further design a feature fusion module to adaptively integrate local texture information and global structural representations. The local branch enhances edges, textures, and fine-grained motion variations, while the global branch captures scene layout, long-range dependencies, and large-scale dynamic changes. For nighttime dynamic reconstruction, directly adding or concatenating these two types of features may introduce redundant responses and weaken the representation of critical regions. Therefore, channel attention and spatial attention are introduced in the fusion stage to emphasize discriminative features related to intensity variation, motion response, and structural recovery.

Specifically, let the local–global fused input feature be denoted as $F_{lg}\in R^{H^{\prime }\times W^{\prime }\times C^{\prime }}$. First, a channel attention map $M_{ca}\in R^{1\times 1\times C^{\prime }}$ is used to model the importance of different feature channels, producing the channel-enhanced feature $F_{ca}$. Then, a spatial attention map $M_{sa}\in R^{H^{\prime }\times W^{\prime }\times 1}$ is applied to model the importance of different spatial locations, generating the spatially enhanced feature $F_{sa}$. This process is formulated as:

(14) $$\left\{\begin{array}{cc} F_{ca} & =M_{ca}\left(F_{lg}\right)⊗F_{lg}\\ F_{sa} & =M_{sa}\left(F_{ca}\right)⊗F_{ca}\\ F^{\prime \prime } & =\mathrm{Swish}\left(\mathrm{BN}\left(\mathrm{Dropout}(F_{sa})\right)\right) \end{array},\right.$$

where ⊗ denotes element-wise multiplication, and $F_{ca}$ and $F_{sa}$ represent the features selected by channel attention and spatial attention, respectively. Channel attention enhances informative channels associated with event responses, intensity variations, and structural recovery, whereas spatial attention focuses on regions with severe blur, significant motion changes, or degraded textures. In this way, the network can better perceive critical regions in nighttime dynamic scenes.

To further improve the robustness and nonlinear representation ability of the fused features, a lightweight regularization and activation block composed of Dropout, BN, and Swish is introduced after attention-based fusion. Dropout randomly suppresses partial feature responses during training, reducing the dependence of the network on local noise or artifacts. BN normalizes feature distributions, stabilizing the training process and accelerating convergence. The Swish activation function provides a smooth nonlinear transformation. Compared with ReLU, which directly truncates negative responses, Swish retains part of the weak negative responses, which is beneficial for feature propagation in weak-texture and low-light regions. Swish is defined as:

(15) $$\mathrm{Swish}\left(c\right)=c\cdot \sigma \left(c\right),\;\sigma \left(c\right)=\frac{1}{1+e^{-c}}.$$

**Figure 4:**
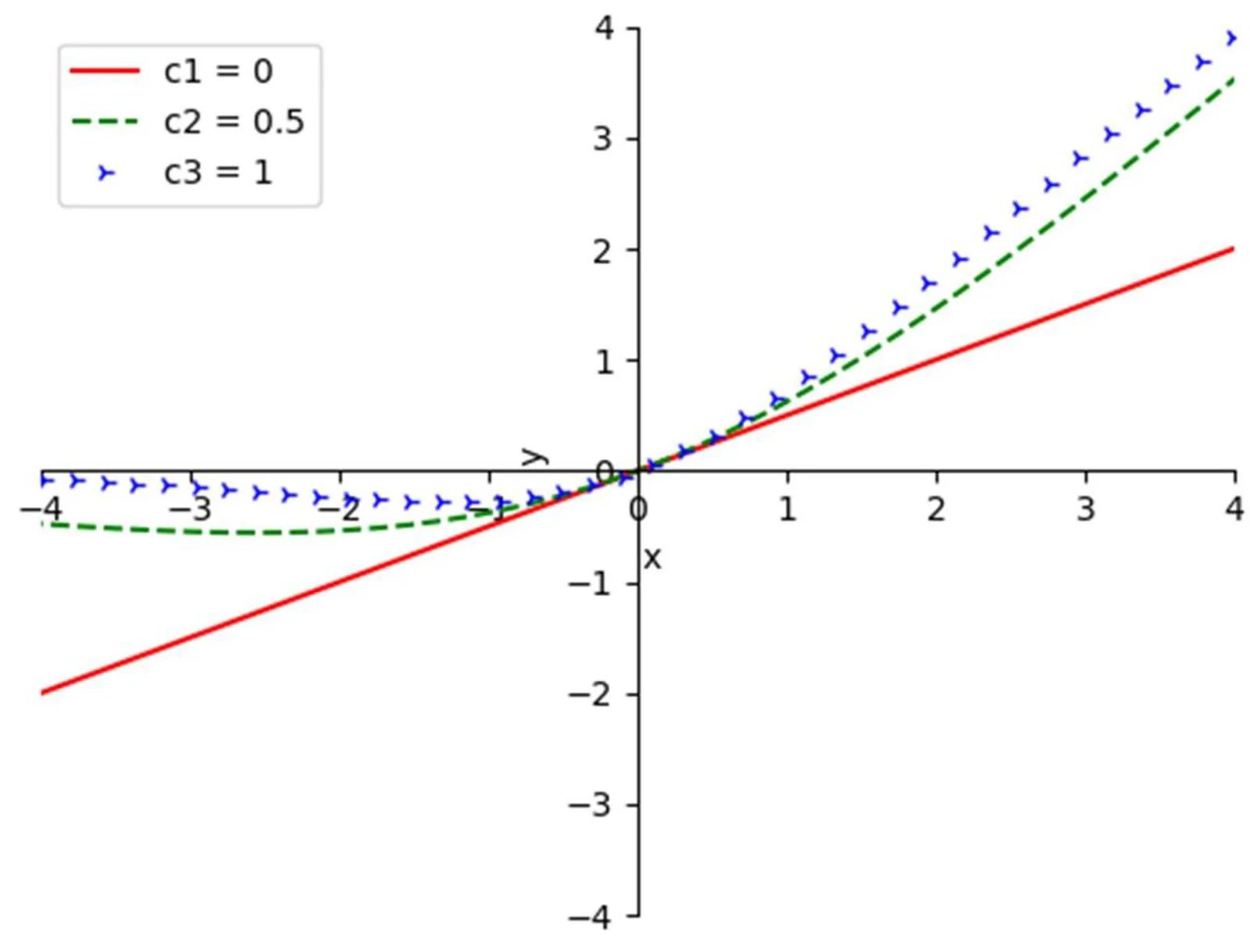
Illustration of the Swish activation function.

As illustrated in Figure 4, Swish maintains continuous and non-zero responses in the negative range, helping to prevent weak signals from being completely suppressed. For nighttime dynamic reconstruction, this property enables the fusion module to preserve subtle structural information in low-light and motion-blurred regions. After channel attention, spatial attention, and lightweight regularization activation, the final output feature $F^{\prime \prime }$ is obtained. This feature integrates local texture details, global structural context, and dynamic-region responses, providing a more stable representation for subsequent image reconstruction. The proposed fusion strategy improves robustness under nighttime dynamic degradation while maintaining low computational complexity.

#### 3.4.4. Encoder, Dual-Branch Bottleneck, and Reconstruction Decoder

The reconstruction backbone follows a four-level U-Net-like encoder–decoder design, while the proposed local–global dual-branch module replaces a conventional single-path bottleneck. The encoder uses four 5×5 convolutions with stride 2 and produces feature maps with 64, 128, 256, and 512 channels. Two residual blocks, each containing two 3×3 convolutions with batch normalization and ReLU activation, first refine the 512-channel bottleneck feature. The refined feature is then processed in parallel by the local and global branches described above. Channel and spatial attention fuse their outputs, followed by Dropout, BN, and Swish to produce $F^{\prime \prime }$.

The decoder restores the spatial resolution through four transposed-convolution stages with output channel dimensions of 256, 128, 64, and 32. Each stage uses a 5×5 transposed convolution with stride 2. The last three stages concatenate the upsampled representation with the encoder feature at the corresponding spatial scale, thereby preserving fine spatial details through skip connections. Finally, the 32-channel decoded feature is concatenated with the original event–frame input and mapped by a 1×1 convolution to the reconstructed output. The encoder–decoder channel configuration follows the U-Net-like backbone of DeblurSR [23], whereas its conventional bottleneck is replaced by the local–global dual-branch and attention-based feature fusion module introduced in this work.

### 3.5. Dual-Constraint Strategy

In nighttime dynamic scenes, low illumination, long exposure, and rapid motion jointly lead to edge degradation, texture loss, and motion blur. For low-light dynamic reconstruction, relying only on pixel-wise reconstruction loss is often insufficient to preserve object contours, scene boundaries, and consistency with the blur-formation process. Therefore, we introduce an edge-alignment constraint and a blur-alignment constraint to improve reconstruction quality from the perspectives of geometric structure and physical imaging consistency, respectively. The edge-alignment constraint enforces consistency between the contour direction of the reconstructed image and the brightness-change direction observed by the event camera, thereby reducing edge distortion and structural misalignment. The blur-alignment constraint requires the degraded version of the reconstructed sharp image to be consistent with the original blurred observation, making the reconstruction process better aligned with the long-exposure imaging mechanism. Together, these two constraints form a dual-supervision strategy that alleviates the lack of geometric consistency and physical constraints in conventional reconstruction methods for nighttime dynamic scenes.

#### 3.5.1. Edge-Alignment Constraint

To obtain the boundary information used by the edge-alignment constraint, the event-count responses from all temporal bins are first aggregated into an event-activity map. Logarithmic compression reduces the influence of isolated high-count responses. The activity map and its event-derived boundary-gradient field are defined as

(16) $$\begin{array}{c} A_{E}\left(x,y\right)=\sum_{b=1}^{B}\log\left(1+E_{b}\left(x,y\right)\right),\;g_{E}\left(x,y\right)=\left[\begin{array}{c} S_{x}*\left(G_{\sigma }*A_{E}\right)\\ S_{y}*\left(G_{\sigma }*A_{E}\right) \end{array}\right] \end{array},$$

where $G_{\sigma }$ is a Gaussian smoothing kernel, $S_{x}$ and $S_{y}$ are fixed Sobel operators, and ∗ denotes convolution. Instead of applying a hard boundary threshold, a continuous confidence weight is used:

(17) $$\begin{array}{c} w_{E}\left(x,y\right)=\frac{∥g_{E}{\left(x,y\right)∥}_{2}}{∥g_{E}{\left(x,y\right)∥}_{2}+\tau } \end{array},$$

where τ>0 suppresses weak responses caused by event noise. Thus, locations with strong and coherent event-boundary responses receive larger weights, whereas empty or noisy regions contribute less to the loss. Let the spatial-gradient field of the reconstructed image be $g_{\hat{L}}\left(x,y\right)={\left[S_{x}*\hat{L},\;S_{y}*\hat{L}\right]}^{T}$. The non-differentiable sign function in the original formulation is removed, and the edge-alignment loss is reformulated using confidence-weighted squared cosine similarity:

(18) $$\begin{array}{c} R_{\mathrm{edge}}=\frac{1}{\sum_{q}w_{E}\left(q\right)+\epsilon }\sum_{q}w_{E}\left(q\right)\left[1-\frac{{\left(g_{\hat{L}}{\left(q\right)}^{T}g_{E}\left(q\right)\right)}^{2}}{\left(∥g_{\hat{L}}{\left(q\right)∥}_{2}^{2}+\epsilon \right)\left(∥g_{E}{\left(q\right)∥}_{2}^{2}+\epsilon \right)}\right] \end{array}.$$

Here, q=(x,y) denotes a spatial location, and ϵ is a small positive constant for numerical stability. Equation (18) is differentiable with respect to $\hat{L}$ and penalizes angular disagreement between reconstructed-image gradients and event-derived boundaries. The squared inner product makes the constraint insensitive to orientation reversal, because event polarity describes temporal brightness changes and does not necessarily determine a unique spatial boundary direction.

#### 3.5.2. Blur-Alignment Constraint

The symbol $K_{E}$ denotes a differentiable event-guided exposure-integration operator rather than a fixed spatial convolution kernel or an additional blur-kernel estimation network. Let $t_{r}$ be the reference time associated with the reconstructed latent sharp image $\hat{L}$, and let ${\left\{t_{m}\right\}}_{m=1}^{M}$ be uniformly sampled times within the exposure interval. The signed event accumulation between $t_{r}$ and $t_{m}$ is defined as

(19) $$\begin{array}{c} P_{m}\left(x,y\right)=\int_{t_{r}}^{t_{m}}e\left(x,y,s\right)\;ds \end{array},$$

where $P_{m}\left(x,y\right)$ is the cumulative signed event response between $t_{r}$ and $t_{m}$. The integral is implemented as the signed sum of event polarities, and changes sign when $t_{m}<t_{r}$. According to the event-generation model, the accumulated logarithmic intensity change is $CP_{m}\left(x,y\right)$, where *C* is the logarithmic contrast threshold introduced in the event-integration model. The corresponding relative intensity factor is therefore $R_{m}\left(x,y\right)=\exp\left(CP_{m}\left(x,y\right)\right)$. The event-guided reblurred observation is then

(20) $$\begin{array}{c} K_{E}\left(\hat{L}\right)=\frac{1}{M}\sum_{m=1}^{M}\hat{L}⊙R_{m} \end{array},$$

where ⊙ denotes element-wise multiplication. Thus, $K_{E}$ approximates temporal exposure integration by combining the reconstructed reference image with the relative brightness changes recorded by the event stream. The operator is differentiable with respect to $\hat{L}$ and introduces no additional learned blur-kernel parameters. The final dual-constraint objective is formulated as

(21) $$\begin{array}{c} L_{\mathrm{total}}=\frac{1}{HW}{∥I_{\mathrm{blur}}-K_{E}\left(\hat{L}\right)∥}_{2}^{2}+\lambda_{e}R_{\mathrm{edge}} \end{array}.$$

The first term enforces consistency between the observed blurred image and the event-guided reblurred reconstruction. The second term aligns the spatial gradient of the reconstructed image with reliable event-derived boundaries. Both terms are differentiable with respect to $\hat{L}$. The weight $\lambda_{e}$ balances blur-formation consistency and edge-structure consistency; the distinct symbols $\lambda_{f}$ and $\lambda_{e}$ are retained to distinguish the fusion weight from the edge-constraint weight.

Through this dual-constraint strategy, the proposed method jointly preserves structural fidelity and imaging plausibility in nighttime dynamic reconstruction. On the one hand, the edge-alignment constraint exploits high-temporal-resolution boundary information from event streams to enhance geometric contour recovery. On the other hand, the blur-alignment constraint makes the reconstruction result consistent with the long-exposure blur-formation process. This design improves reconstruction robustness under complex low-light dynamic conditions.

## 4. Experimental Validation

### 4.1. Datasets and Event Data Preparation

We evaluate the proposed method on two public benchmark datasets, namely, REDS [24] and HQF [25]. Neither dataset was captured from a real UAV platform; consequently, the experiments evaluate benchmark-level performance in low-light dynamic visual reconstruction and event-assisted image restoration rather than UAV-specific operational performance. The datasets contain key factors such as dynamic scenes, blur degradation, event responses, and video restoration settings. These factors are technically relevant to potential nighttime UAV applications, but validation using data captured during actual UAV flight is still required:

- **REDS:** This is a large-scale video restoration dataset introduced in the NTIRE 2019 video restoration challenge. It is commonly divided into training, validation, and test sets with a ratio of 240/30/30. Each video sequence contains approximately 100 frames, and the original sharp frames have a resolution of 720×1280. REDS is widely used for image deblurring, video restoration, and super-resolution tasks. Because REDS contains conventional frames but no event-camera measurements, the corresponding raw events and blurred observations are synthesized following the ESIM-based data-generation protocol adopted by DeblurSR [23]. The original 120-fps sharp sequences are converted to grayscale, resized to 180×240, and temporally interpolated to 960 fps using SepConv-SloMo. The interpolated intensity sequence is then processed by ESIM. The exposure duration is set to $T_{\exp}=120$ ms. ESIM permits the two polarities to be configured separately; in our synthetic REDS experiments, their nominal magnitudes are set identically as $C_{+}=C_{-}=0.18$, with positive and negative threshold standard deviations both set to 0.03. Accordingly, the negative triggering boundary is $-C_{-}=-0.18$, rather than +0.18. Log-intensity simulation is enabled with $\epsilon_{\log}=0.01$. These values are simulation settings rather than thresholds calibrated from a physical sensor.
  For the *k*th blurred frame with central timestamp $t_{k}^{B}$, the exposure boundaries are defined as
  (22) $$t_{k}^{s}=t_{k}^{B}-\frac{T_{\exp}}{2},\;t_{k}^{e}=t_{k}^{B}+\frac{T_{\exp}}{2},$$
  and hence $\left[t_{k}^{s},t_{k}^{e}\right]=\left[t_{k}^{B}-0.06,\;t_{k}^{B}+0.06\right]$ s. The blurred observation is formed by integrating the interpolated sharp frames within this interval, and only events satisfying $t_{i}\in \left[t_{k}^{s},t_{k}^{e}\right]$ are associated with that observation. The same exposure interval is divided into B=13 temporal bins. Instead of using the 26-channel polarity-separated representation of DeblurSR, the proposed method calculates the event count, average polarity response, and average normalized timestamp in every bin, yielding the D=3B=39 voxel representation defined in Section 3.2. Event timestamps are normalized within the exposure window as
  (23) $${\tilde{t}}_{i}=\frac{t_{i}-t_{k}^{s}}{t_{k}^{e}-t_{k}^{s}}\in \left[0,1\right].$$
  Because the proposed network reconstructs one latent sharp image rather than a sharp-frame sequence, the interpolated sharp frame at $t_{k}^{B}$, or the temporally nearest available frame, is used as the ground-truth target. Consequently, the blurred input, event voxel, and ground-truth sharp frame share the same spatial coordinates and exposure-centered temporal reference. During training, the same 160×160 spatial crop is applied to all three components to preserve pixel-wise alignment.
- **HQF:** This is a real event-camera dataset captured by DAVIS240C sensors, containing real event streams and corresponding image frames. The dataset has a spatial resolution of 240 × 180 and includes event data under different scenes, motion patterns, and noise conditions. It is commonly used to evaluate event-based image reconstruction, event-assisted deblurring, event-based video restoration, and event optical flow methods. Compared with synthetic blurred data, HQF better reflects the asynchronous response, noise disturbance, and spatiotemporal sparsity of event cameras in real imaging processes. Therefore, it is suitable for evaluating the robustness of event–frame fusion methods on real event data. In this paper, we further visualize reconstruction results from three scene categories, including persons, objects, and letters, to intuitively demonstrate the reconstruction capability of the proposed method under different structural patterns and motion conditions. For HQF, the recorded events are used directly. Their triggering thresholds are determined by the DAVIS240C sensor settings during acquisition and are not re-estimated or replaced by the ESIM thresholds used for REDS.

### 4.2. Evaluation Metrics

We adopt the mean squared error (MSE) [26], Peak Signal-to-Noise Ratio (PSNR) [27], and Structural Similarity Index Measure (SSIM) [28] as the main image quality assessment metrics to quantitatively compare the proposed method with existing event-based blurred image reconstruction methods. The combination of these three metrics provides a comprehensive quantitative evaluation of nighttime dynamic reconstruction quality on the selected benchmarks. We adopt the MSE, PSNR, and SSIM as the main image quality assessment metrics to quantitatively compare the proposed method with existing event-based blurred image reconstruction methods. These metrics evaluate the reconstruction results from pixel-wise error, signal fidelity, and perceptual structural similarity, respectively, making them suitable for nighttime dynamic visual enhancement and event-assisted image restoration tasks:

- **MSE:** For each test image *n*, the pixel-wise mean squared error is first calculated, and the reported dataset-level MSE is the arithmetic mean of the per-image values:
  (24) $$\begin{array}{c} \mathrm{MSE}=\frac{1}{N}\sum_{n=1}^{N}{\mathrm{MSE}}_{n},\;{\mathrm{MSE}}_{n}=\frac{1}{HWC}\sum_{x=1}^{W}\sum_{y=1}^{H}\sum_{c=1}^{C}{\left({\hat{I}}_{n}\left(x,y,c\right)-I_{n}\left(x,y,c\right)\right)}^{2} \end{array},$$
  where *N* is the number of test images, *H*, *W*, and *C* denote the image height, width, and number of channels, and ${\hat{I}}_{n}$ and $I_{n}$ are the reconstructed and reference images, respectively. A lower MSE indicates a smaller numerical reconstruction error.
- **PSNR:** The PSNR is also calculated independently for every test image and is subsequently averaged over the dataset:
  (25) $$\begin{array}{c} \mathrm{PSNR}=\frac{1}{N}\sum_{n=1}^{N}10\log_{10}\left(\frac{MAX_{I}^{2}}{{\mathrm{MSE}}_{n}}\right) \end{array},$$
  where $MAX_{I}$ denotes the maximum image intensity. We use $MAX_{I}=1$ for images normalized to [0,1]. Because the logarithm is nonlinear, the mean PSNR is generally not equal to $10\log_{10}\left(MAX_{I}^{2}/\mathrm{MSE}\right)$, where the MSE is the dataset-level mean reported in the table. The tabulated MSE and PSNR values are therefore independently averaged per-image statistics and should not be directly converted into one another.
- **SSIM:** The SSIM evaluates the local structural similarity between the reconstructed image and the reference image from luminance, contrast, and structural perspectives. It is defined as:
  (26) $$\mathrm{SSIM}\left(x,y\right)=\frac{\left(2\mu_{x}\mu_{y}+C_{1}\right)\left(2{\sigma_{x}}_{y}+C_{2}\right)}{\left(\mu_{x}^{2}+\mu_{y}^{2}+C_{1}\right)\left(\sigma_{x}^{2}+\sigma_{y}^{2}+C_{2}\right)},$$
  where $\mu_{\hat{I}}$ and $\mu_{I}$ denote the local means of the reconstructed and reference images, respectively. $\sigma_{\hat{I}}^{2}$ and $\sigma_{I}^{2}$ are their local variances, and $\sigma_{\hat{I}I}$ is the local covariance. $C_{1}$ and $C_{2}$ are stabilization constants, usually defined as $C_{1}={\left(K_{1}L\right)}^{2}$ and $C_{2}={\left(K_{2}L\right)}^{2}$, where *L* is the image dynamic range, $K_{1}=0.01$, and $K_{2}=0.03$. The SSIM typically ranges from 0 to 1, with a higher value indicating better structural similarity.

Overall, the MSE and PSNR mainly reflect pixel-wise numerical error and signal fidelity, while the SSIM emphasizes perceptual structural consistency. The combination of these three metrics provides a comprehensive quantitative evaluation of nighttime dynamic reconstruction quality on REDS and HQF.

### 4.3. Experimental Platform and Parameter Settings

All experiments were implemented in PyTorch 1.12.1 and conducted on a single NVIDIA A100 GPU. Training samples were randomly cropped into 160×160 patches, and the batch size was set to four. The network was trained for 50 epochs with an initial learning rate of $1\times 10^{-4}$. A step-wise learning-rate schedule was used, reducing the learning rate by 50% after the 20th and 40th epochs. The spatial convolution kernel size was set to 3×3, enabling local spatial structures to be extracted with low computational overhead. The number of bins in the gradient histogram was set to 26 to provide a more detailed description of edge orientations and local structural variations. The dimensions of both image embeddings and coordinate embeddings were set to 256, which enhances the joint representation of image intensity information, spatial positional cues, and event-based temporal features. These parameter settings provide a favorable balance between reconstruction accuracy and computational efficiency for the evaluated nighttime dynamic reconstruction task.

### 4.4. Quantitative Analysis

As shown in Table 1, the proposed method demonstrates strong motion-blurred image reconstruction capability on both benchmark datasets. Compared with DeblurSR on HQF, the proposed method reduces the MSE from 0.162 to 0.138, corresponding to a relative reduction of 14.81%, while improving the PSNR by 1.603 dB and the SSIM by 7.94%. On REDS, it reduces the MSE from 0.100 to 0.090, corresponding to a 10.00% reduction, while improving the PSNR by 1.053 dB and the SSIM by 2.68%. These results indicate that the proposed method not only effectively reduces pixel-wise reconstruction errors but also achieves better structural similarity and visual fidelity. Since HQF contains real event streams and REDS includes complex dynamic blur, the consistent improvements on both datasets demonstrate the robustness of the proposed method against real event noise, dynamic blur, and structural degradation.

#### 4.4.1. Parameter-Sensitivity Analysis

To avoid test-set tuning, $\lambda_{f}$ and $\lambda_{e}$ were selected exclusively on the official REDS validation split, which contains 30 video sequences of approximately 100 frames each. We evaluated the Cartesian grid $\lambda_{f}\in \left\{0.3,0.5,0.8\right\}$ and $\lambda_{e}\in \left\{0.01,0.05,0.10\right\}$, giving nine parameter combinations. All other network architecture, training, input-resolution, learning-rate, and preprocessing settings were kept unchanged. The PSNR was used as the primary selection metric, with the MSE and SSIM serving as complementary error and structural-fidelity measures. As summarized in Table 2, the selected values were $\lambda_{f}=0.5$ and $\lambda_{e}=0.05$. These values were then fixed for evaluation on HQF without additional tuning; the HQF columns therefore assess cross-dataset stability rather than participate in parameter selection.

Table 2 shows a consistent non-monotonic response to both weights. For every tested value of $\lambda_{f}$, the intermediate edge-constraint weight $\lambda_{e}=0.05$ outperforms 0.01 and 0.10 on all three REDS validation metrics. An overly weak edge constraint therefore provides insufficient structural guidance, whereas an overly strong constraint may overemphasize event boundaries and restrict intensity reconstruction. Similarly, $\lambda_{f}=0.5$ consistently outperforms 0.3 and 0.8 at each matched value of $\lambda_{e}$, showing that neither event-dominant nor frame-dominant fusion is optimal. The balanced choice of $\lambda_{f}=0.5$ and $\lambda_{e}=0.05$ achieves the lowest REDS validation MSE of 0.090, the highest PSNR of 24.079 dB, and the highest SSIM of 0.882. Compared with the other eight settings, this choice improves the PSNR by 0.54% to 2.25% and the SSIM by 0.34% to 1.85%, while reducing the MSE by 2.17% to 10.00%. After the weights selected on REDS were fixed, the same choice also produced the best HQF performance, with an MSE of 0.138, a PSNR of 19.805 dB, and an SSIM of 0.748. The consistent performance on synthetic dynamic-blur data and real event data supports the reasonableness and cross-dataset robustness of the selected parameters, while HQF was not used for retuning.

#### 4.4.2. Component-Wise Ablation Study

To separately quantify the contribution of each proposed component, we conducted a progressive ablation study under a strictly controlled protocol. The baseline was constructed using the basic reconstruction backbone adapted from DeblurSR, while excluding the proposed multi-channel voxel representation, local–global dual-branch structure, channel attention, spatial attention, edge-alignment loss, and blur-alignment loss. All configurations were trained and evaluated using the same dataset splits, event-generation procedure, input resolution, optimizer, learning-rate schedule, number of epochs, and evaluation metrics. Starting from the baseline, the multi-channel voxel representation, local–global reconstruction structure, channel attention, spatial attention, edge-alignment loss, and blur-alignment loss were introduced sequentially. Therefore, the difference between two adjacent rows measures the incremental contribution of the newly introduced component under otherwise unchanged settings.

In Table 3, MCV denotes the multi-channel voxel representation, L–G denotes the local–global dual-branch reconstruction structure, CA denotes channel attention, and SA denotes spatial attention. The reconstruction performance improves consistently as the proposed components are progressively introduced. Incorporating MCV increases the PSNR from 23.026 to 23.214 dB on REDS and from 18.202 to 18.548 dB on HQF. The larger improvement on HQF indicates that jointly encoding event count, polarity response, normalized timestamps, and blurred-frame intensity priors is particularly beneficial when processing real event streams.

Introducing the local–global dual-branch reconstruction structure further improves the PSNR to 23.438 dB on REDS and 18.896 dB on HQF, supporting the complementary roles of local texture recovery and global structural modeling. Channel attention and spatial attention provide separate and consistent improvements. Channel attention increases the PSNR by 0.174 dB on REDS and 0.236 dB on HQF, while spatial attention provides additional gains of 0.136 and 0.194 dB, respectively. These results show that channel-wise feature recalibration and spatially selective enhancement make distinct contributions.

The edge-alignment loss further improves the PSNR from 23.748 to 23.902 dB on REDS and from 19.326 to 19.552 dB on HQF, demonstrating the benefit of event-derived boundary supervision. Finally, adding the blur-alignment loss produces the full model, yielding 24.079 dB on REDS and 19.805 dB on HQF. Relative to the baseline, the complete method improves the PSNR by 1.053 dB on REDS and 1.603 dB on HQF. The monotonic improvement across all configurations demonstrates that the final performance arises from the complementary contributions of the representation, reconstruction architecture, attention mechanisms, and dual-alignment objective rather than being dominated by a single component.

The progressive experiment in Table 3 measures the incremental contribution of each component relative to the preceding configuration. As a complementary analysis, Table 4 removes one principal component at a time from the full model to examine whether its contribution remains necessary when all other components are present.

All investigated components contribute positively to the final reconstruction performance. Removing event–frame fusion causes the largest degradation: the PSNR decreases from 24.079 to 22.694 dB on REDS and from 19.805 to 18.156 dB on HQF, while the SSIM decreases from 0.882 to 0.845 and from 0.748 to 0.695, respectively. This confirms that complementary frame intensity priors and event-based temporal responses form the principal basis of the reconstruction framework. Removing the local–global branch decreases the PSNR by 0.892 dB on REDS and 1.081 dB on HQF, demonstrating the importance of jointly modeling local textures and global structural context. The attention module produces a smaller but consistent improvement on both datasets, indicating that channel and spatial attention refine informative feature selection rather than acting as the sole source of the performance gain.

The two constraints provide complementary supervision. Without the edge constraint, the HQF SSIM decreases from 0.748 to 0.733, showing that event-derived boundary guidance primarily benefits structural preservation. Without the blur constraint, the HQF MSE increases from 0.138 to 0.146, and the PSNR decreases from 19.805 to 19.311 dB, indicating that exposure-consistency supervision contributes more directly to pixel-level fidelity. The full model achieves the best result for every metric on both datasets, supporting the combined design rather than any single component alone. The progressive results in Table 3 and the leave-one-component-out results in Table 4 provide complementary evidence. The former shows how performance accumulates as individual components are introduced, whereas the latter confirms that removing any principal component from the complete system causes performance degradation. Together, the two analyses demonstrate both the independent incremental value and the full-model necessity of the proposed components.

#### 4.4.3. Model Complexity and Runtime Analysis

We compare the model size, computational cost, inference latency, and throughput with DeblurSR under a unified evaluation protocol. Parameter counts and GFLOPs are calculated for a single 160×160 input with a batch size of one. Runtime is measured on a single NVIDIA A100 GPU with a batch size of four and is reported as the average processing time per sample. Equivalent throughput is calculated from the measured per-sample latency as FPS=1000/Time. Both methods are evaluated using the same software environment, input resolution, batch size, and timing procedure.

As shown in Table 5, the proposed model contains 20.017 M trainable parameters, compared with 20.002 M for DeblurSR. The difference is only 0.015 M, corresponding to approximately 0.075%, indicating that the proposed feature-processing modules introduce little additional parameter storage. The computational cost increases from 163.06 to 184.96 GFLOPs, while the measured processing time increases from 21.183 to 23.756 ms per sample under the stated batched A100 protocol. These measurements correspond to equivalent throughputs of 47.21 and 42.09 FPS, respectively. The moderate computational increase arises from the local–global feature processing, attention-based feature selection, and richer multi-channel event representation. In return, the proposed model consistently improves the MSE, PSNR, and SSIM on both REDS and HQF.

Accordingly, “lightweight” in this work refers specifically to the parameter-efficient construction of the proposed feature-processing modules rather than to a lower overall FLOP count or latency than DeblurSR. The proposed method does not claim an absolute computational advantage over DeblurSR. Instead, the complete model maintains an almost identical parameter footprint while providing additional representation capacity and higher reconstruction accuracy, thereby demonstrating a favorable accuracy–complexity trade-off. Peak GPU memory and total training time are not reported because these quantities were not recorded for both methods under an identical controlled protocol; reporting values obtained under different hardware, software, or workloads could lead to an unfair comparison.

The runtime measurements were obtained on a server-grade NVIDIA A100 GPU rather than an embedded onboard platform. They provide a reproducible computational-efficiency reference but do not constitute direct validation of real-time UAV onboard deployment. Future work will evaluate single-frame latency, memory consumption, and power efficiency on embedded platforms such as NVIDIA Jetson devices, together with hardware-aware model compression and mixed-precision inference.

#### 4.4.4. Methodological Comparison and Novelty Positioning

To distinguish the proposed technical contribution from established event-guided reconstruction techniques, Table 6 compares representative methods in terms of input representation, architecture or inference mechanism, supervision, and distinctive contribution. The comparison focuses on methodological differences rather than treating individual operators as independent innovations.

Table 6 shows that the proposed method is not distinguished by the isolated use of voxel grids, attention operations, depthwise separable convolution, Swish activation, or structural losses. These operations are established building blocks. The methodological distinction lies in how they are organized around a different reconstruction objective and representation. EDI directly solves an event-based physical integration model, whereas eSL-Net unfolds a sparse-learning formulation. EventSR reconstructs and super-resolves intensity images from pure events through a multi-stage adversarial pipeline. E-CIR and DeblurSR recover temporally continuous or high-frame-rate outputs through parametric time-to-intensity representations. In contrast, the present method targets single-frame reconstruction under combined low illumination, dynamic blur, and unreliable event responses.

For this purpose, the proposed representation preserves three complementary event statistics within each of 13 temporal bins and introduces normalized blurred-frame intensity before feature extraction. The resulting 39-channel representation is processed through coordinated local and global pathways, while channel and spatial attention select informative responses. The reconstructed image is further constrained by a differentiable event-boundary alignment term and an event-guided reblur operator. Therefore, the contribution is the task-specific integration of representation, reconstruction, and supervision rather than the invention of the individual operators.

**Figure 5:**
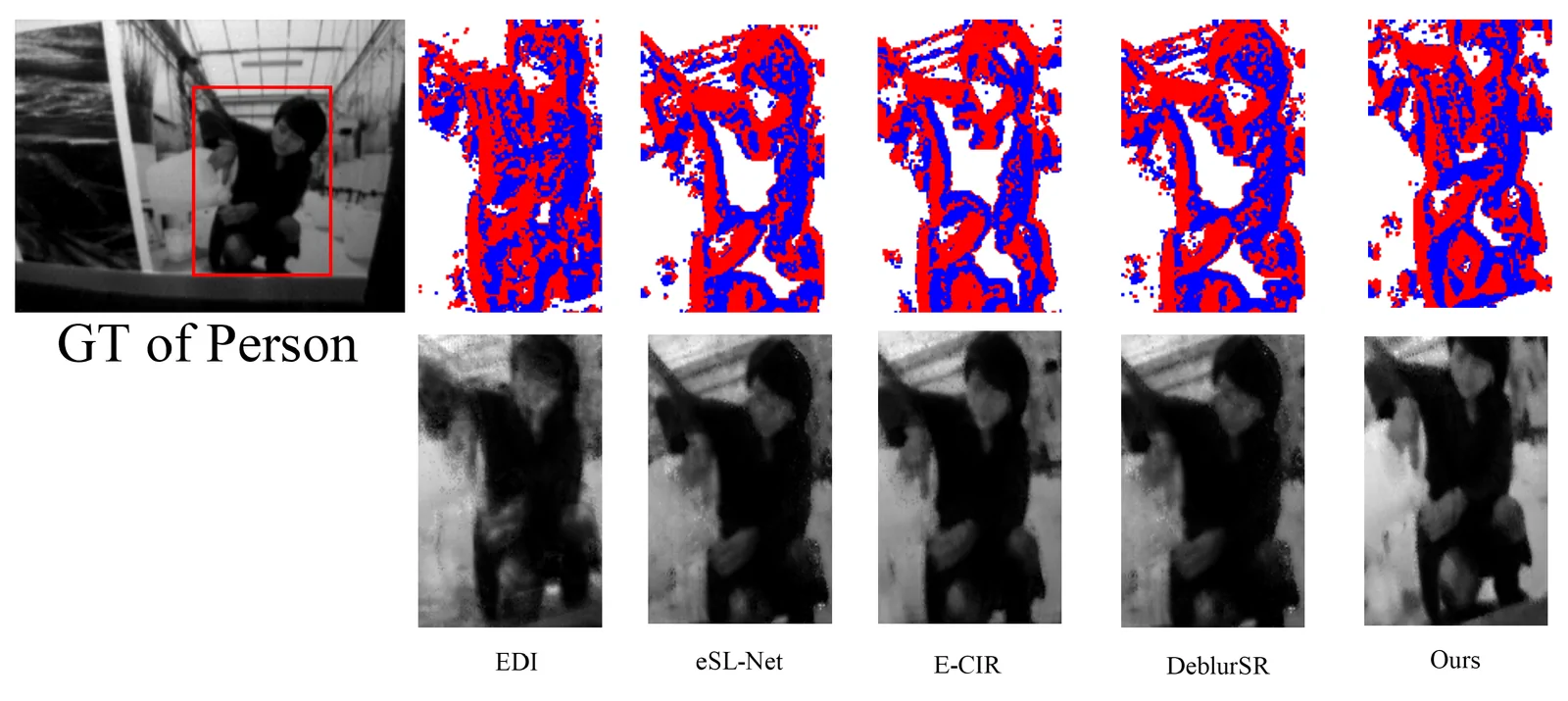
Local-detailreconstruction comparison in a dynamic person scene. The **left** image shows the input blurred frame, and the red box marks the dynamic region of interest. The **upper** row presents the corresponding event-response or boundary-aware representations of different methods, while the **lower** row shows the reconstructed local results. This scene is mainly used to evaluate contour recovery, boundary continuity, and noise suppression under non-rigid human motion.

In the person scene as in Figure 5, motion blur is mainly concentrated around the human contour, arm boundary, and torso region, representing a typical non-rigid motion degradation case. As shown in the figure, although EDI and eSL-Net can recover part of the human structure with the aid of event information, their reconstructions still exhibit noticeable boundary diffusion and local noise. The human contour remains incomplete, especially around the arm and head regions, where blur residuals are evident. E-CIR improves the overall structure to some extent, but local boundaries still show discontinuities. DeblurSR restores relatively smooth contours, yet edge softening can still be observed in local texture regions.

In contrast, the proposed method achieves better recovery of human contours, arm boundaries, and body posture structures. The boundaries in dynamic regions are clearer and more continuous, while background interference and noise artifacts are more effectively suppressed. This superiority mainly comes from two aspects. First, the proposed multi-channel event voxel representation jointly models event count, event intensity, timestamp distribution, and blurred-frame intensity priors, enabling the network to better distinguish dynamic human boundaries from the surrounding background. Second, the edge-alignment constraint guides the gradient direction of the reconstructed image using event-boundary directions, thereby improving the geometric consistency of human contour recovery. These results indicate improved robustness for reconstructing non-rigid motion and preserving local boundaries under the nighttime degradation conditions represented in HQF.

**Figure 6:**
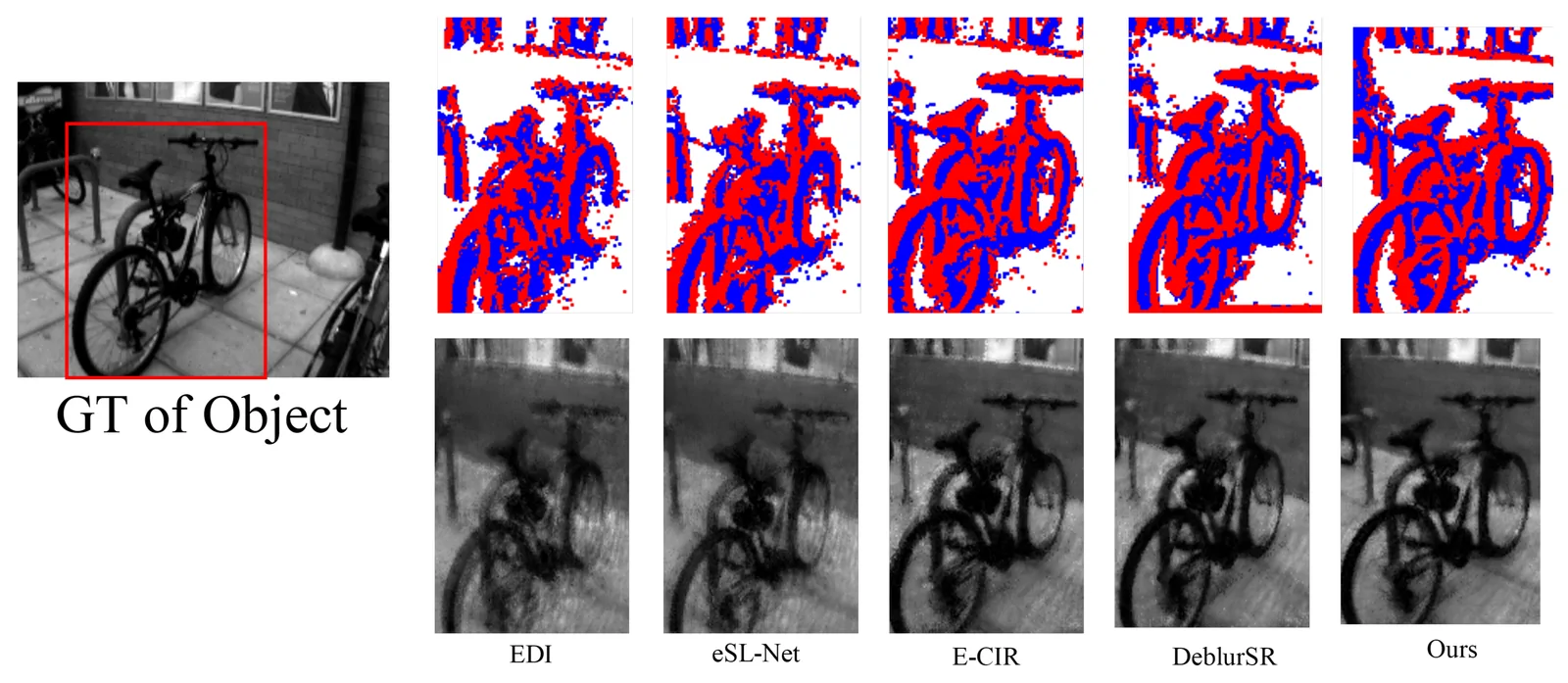
Reconstruction comparison of fine structures in a bicycle scene. The **left** image shows the input blurred frame, and the red box highlights the bicycle region. The **upper** row shows the response representations of different methods, and the **lower** row presents the local reconstruction results. This scene mainly evaluates the ability of different methods to recover thin structures, contour closure, and complex local geometric relationships.

In the bicycle scene, as shown in Figure 6, the image contains numerous thin structures and complex geometric relationships, such as wheel contours, frame junctions, and handlebar regions. Such details impose a higher requirement on nighttime dynamic reconstruction. From the results, EDI and eSL-Net can recover the rough shape of the bicycle at a coarse scale, but they suffer from evident boundary blur and structural adhesion around the rim, frame, and thin-line regions, leading to inaccurate local geometry. E-CIR performs better than the first two methods in restoring the main structure, yet it still shows contour discontinuity and incomplete local details. DeblurSR yields relatively stable overall visual quality, but texture loss and edge smoothing are still noticeable in fine-structure regions.

The proposed method shows more pronounced advantages in this scene. The wheel edges are more complete, thin structures such as the frame and front fork are clearer, and the overall geometric layout is closer to the true structure. This can be mainly attributed to the proposed local–global dual-branch feature fusion network, which jointly considers local-detail restoration and global structural consistency. Specifically, the local branch strengthens the representation of rims, spoke-related regions, and fine boundary details, while the global branch maintains the overall frame layout and contour closure through long-range dependency modeling. Furthermore, the blur-alignment constraint ensures that the reconstruction remains consistent with the original blurred observation while being visually sharper, thereby reducing structural distortion caused by over-enhancement. These results demonstrate improved recovery of thin structures, contour closure, and complex geometry under dynamic blur.

**Figure 7:**
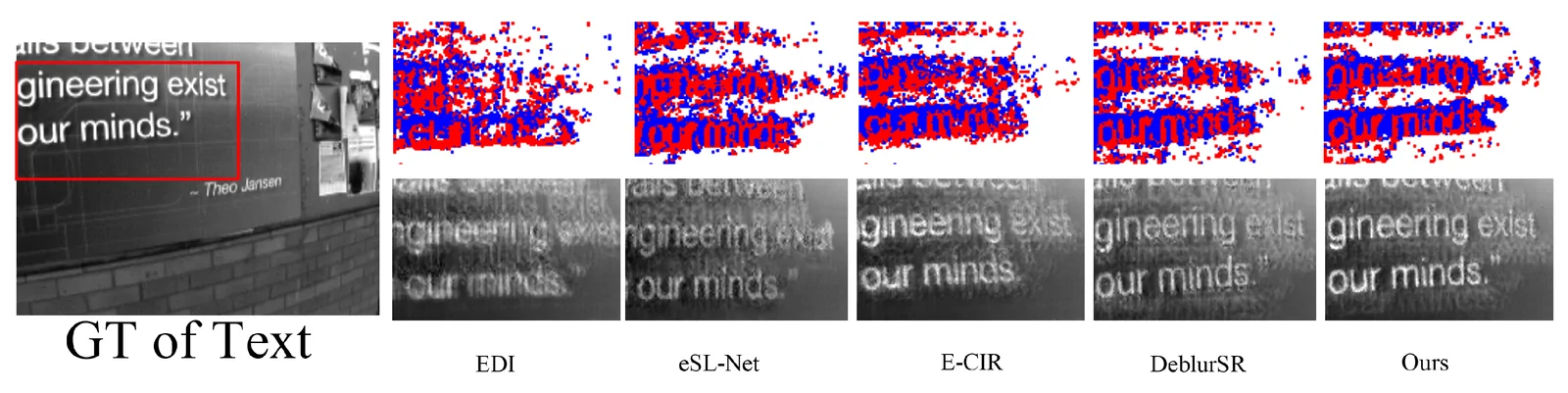
Readability-oriented reconstruction comparison in a text scene. The **left** image shows the input blurred frame, and the red box highlights the text region. The **upper** row shows the response representations of different methods, and the **lower** row presents the local reconstruction results for the text region. This scene is used to evaluate the capability of different methods in restoring high-frequency details, preserving stroke continuity, and improving semantic readability.

As shown in Figure 7, the text scene imposes requirements different from the previous two categories. Its core objective is to recover stroke boundaries, improve inter-character separation, and enhance overall readability. As observed from the figure, EDI and eSL-Net suffer from evident blur residuals and granular noise in the text region. Character strokes are discontinuous, and some local words are difficult to recognize. E-CIR improves the contour recovery of some characters, but the stroke edges remain insufficiently sharp, and the local contrast is still weak. DeblurSR restores part of the text structure, yet noticeable blur remains in character details and line boundaries.

The proposed method demonstrates stronger high-frequency detail recovery in the text scene. Character edges are clearer, stroke continuity is better preserved, and the overall readability is significantly improved compared with other methods. This advantage mainly stems from the proposed event–frame fusion mechanism and the attention-driven feature fusion strategy. On the one hand, the blurred frame provides stable spatial intensity priors, while the event stream supplies high-temporal-resolution cues for text-boundary changes. Their combination is beneficial for reconstructing fine-grained stroke structures. On the other hand, the channel attention and spatial attention modules in the fusion stage more effectively emphasize text edges and high-frequency response regions, enhancing the perception of critical character information. In addition, the Swish activation and lightweight regularization module help preserve weak-response details in low-light regions and reduce character breakage and background noise interference. These results indicate that the proposed method improves stroke continuity and information readability under low-light dynamic degradation.

In summary, the three qualitative experiments on the HQF dataset systematically validate the proposed method from three aspects: non-rigid target contour recovery, complex geometric structure reconstruction, and high-frequency detail readability. The analysis relates these improvements to the multi-channel event voxel representation, local–global dual-branch network, attention-based fusion mechanism, and the edge- and blur-alignment constraints. The conclusions drawn here are restricted to the evaluated benchmark scenes.

### 4.5. Qualitative Error and Edge-Consistency Analysis

**Figure 8:**
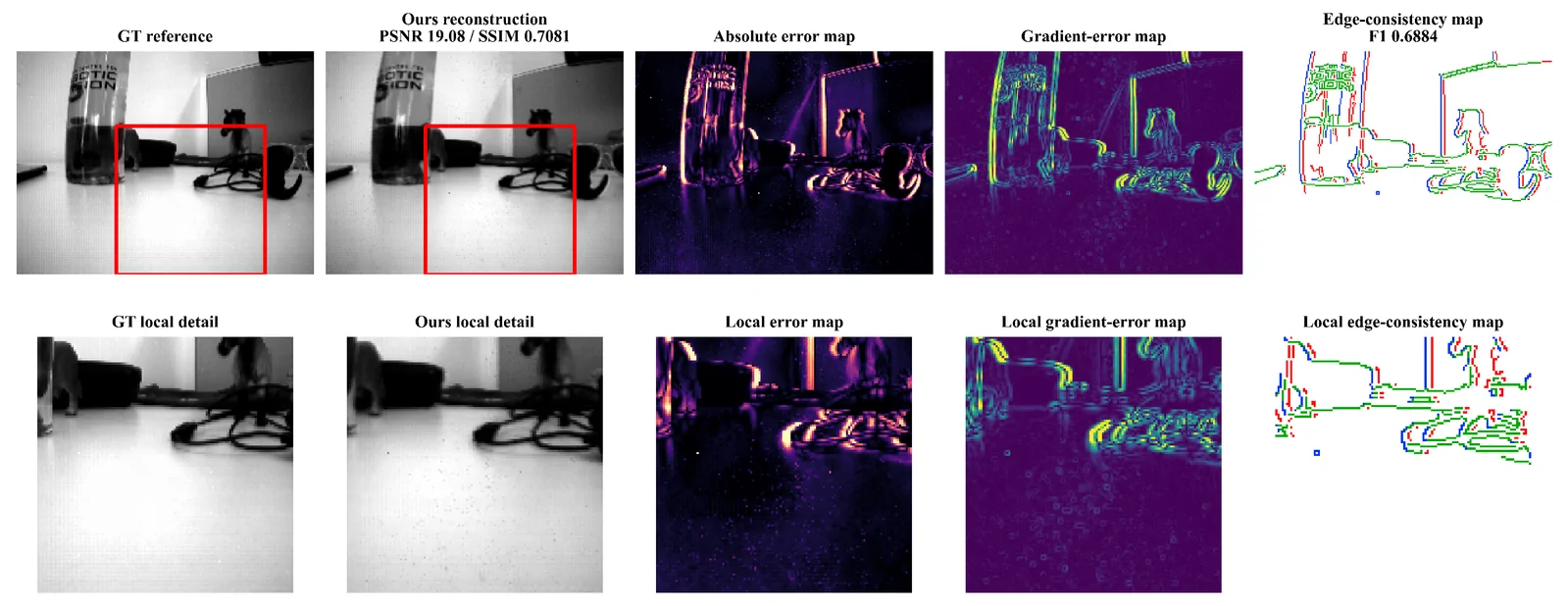
Qualitative visualization of local reconstruction details, absolute errors, gradient errors, and edge consistency on HQF. The first row shows the ground truth, full-model reconstruction, absolute error, gradient error, and edge consistency; the second row shows the corresponding local details. Green, red, and blue denote matched edges, missed ground-truth edges, and falsely reconstructed edges, respectively. Component-wise quantitative results are reported separately in Table 3 and Table 4.

As shown in Figure 8, the proposed method maintains good consistency with GT in terms of global intensity distribution and main structural recovery. In the Ours reconstruction result, the target objects, tabletop structures, and background regions are visually stable, indicating that the proposed event–frame fusion strategy effectively combines spatial intensity priors from the blurred frame with high-temporal-resolution motion cues from the event stream. This provides a more reliable spatiotemporal input for nighttime dynamic reconstruction.

The absolute error map shows that the proposed method produces relatively low errors in large flat and weak-texture regions, while higher errors are mainly concentrated around object boundaries, reflective regions, and thin structures. This indicates that the proposed method can recover global intensity and main structures effectively, whereas high-frequency edges remain challenging in nighttime dynamic reconstruction. This observation also supports the effectiveness of the multi-channel event voxel representation. By jointly encoding event count, event intensity, timestamp distribution, and blurred-frame intensity, the model better exploits the complementarity between event and frame cameras. This design reduces local information loss caused by low illumination and motion blur.

The gradient-error map further shows that structural deviations mainly appear around bottle boundaries, object contours, and line-like structures. Compared with methods that rely only on pixel-wise reconstruction constraints, the proposed local–global dual-branch feature fusion network enables the model to recover edge and texture details through the local branch while maintaining holistic structure and long-range contextual consistency through the global branch. Therefore, the proposed method reduces pixel-wise errors while preserving more stable structural representations. The edge-consistency map directly demonstrates the effect of the edge-alignment constraint. A large number of green edges indicate that the reconstructed result is well aligned with the main GT boundaries, while the red and blue regions are relatively localized, showing that missing edges and false edges are effectively suppressed. This demonstrates that the edge-alignment constraint can guide the model to recover more accurate geometric contours and reduce edge drift, structural misalignment, and local artifacts commonly observed in nighttime dynamic scenes.

The local-detail region further confirms this advantage. Our local detail remains highly consistent with GT local detail in tabletop regions, object boundaries, and local structures. The local error map and local gradient-error map show that errors are mainly distributed around high-frequency variations, while most regions remain stable. The local edge-consistency map further confirms that the proposed method preserves local geometric structures well. Overall, Figure 8 provides a qualitative visualization of the full model in terms of local reconstruction details, absolute errors, gradient errors, and edge consistency. It is intended to illustrate the spatial distribution of the remaining reconstruction errors rather than to serve as a component-wise ablation experiment. The individual contributions of the proposed components are evaluated quantitatively through the progressive and leave-one-component-out experiments reported in Table 3 and Table 4.

Therefore, this qualitative result demonstrates that the proposed method not only achieves visually favorable reconstruction but also exhibits greater stability in error distribution, gradient structure, and edge consistency. These benchmark results support the method’s effectiveness for nighttime dynamic image reconstruction without implying UAV-specific operational validation.

### 4.6. Technical Relevance to Nighttime UAV Imaging

Nighttime UAV imaging frequently involves weak illumination, exposure-induced motion blur, rapid brightness variation, platform vibration, and noisy or sparse event responses. These degradation factors overlap with the technical problems addressed by the proposed event–frame fusion representation and its edge- and blur-alignment constraints. The results on REDS and HQF therefore provide evidence that the method can handle several degradation mechanisms that are relevant to nighttime UAV imaging. This relevance is technical rather than operational: REDS contains synthetically generated blur and HQF was not acquired from a UAV in flight, so neither dataset represents the complete distribution of airborne nighttime imagery. In particular, the present experiments do not evaluate flight-induced vibration, rapid attitude changes, altitude-dependent viewpoints, embedded-device constraints, or downstream UAV tasks. Accordingly, no claim of demonstrated effectiveness in UAV monitoring, inspection, search and rescue, or onboard perception is made. Such claims require synchronized frame–event data collected during real nighttime flights and platform-level evaluation.

## 5. Conclusions and Discussion

This paper addresses image reconstruction in nighttime dynamic scenes. We propose an event–frame fusion method for robust visual reconstruction. The method targets visual degradation caused by low illumination, long exposure, motion blur, and event noise. It jointly exploits spatial intensity information from frame images and high-temporal-resolution motion cues from event streams. This design enables more stable reconstruction in nighttime dynamic scenes. Because the same degradation factors can arise in nighttime UAV imaging, this study has technical relevance to that setting; however, the present benchmark experiments do not constitute validation on a real UAV platform.

The main contribution is the coordinated design of three task-specific elements rather than the individual use of established operators. First, the 39-channel event–frame representation jointly encodes event count, average polarity response, normalized timestamp, and blurred-frame intensity priors. Second, the representation-aware local–global pathway coordinates fine-texture recovery, holistic structural modeling, and channel–spatial feature selection. Third, the differentiable dual-alignment objective combines reliable event-boundary guidance with event-guided reblurring to constrain geometric and exposure-formation consistency.

Experimental results show that the proposed method achieves better MSE, PSNR, and SSIM performance than existing methods on the HQF and REDS datasets. Qualitative results further show that the proposed method restores sharper edges, more complete local textures, and more stable global structures in person, object, and text scenes. Error maps, gradient-error maps, and edge-consistency maps also demonstrate that the proposed method reduces pixel-wise errors and improves structural edge consistency. These results verify the effectiveness of event–frame fusion, multi-channel voxel representation, local–global feature modeling, and the dual-constraint strategy.

Despite these promising results, several aspects remain to be improved. The present evidence is limited to the REDS and HQF benchmarks, neither of which consists of data captured during actual UAV flight. Therefore, the results demonstrate benchmark-level nighttime dynamic reconstruction rather than UAV-specific operational effectiveness. Systematic evaluation on real nighttime UAV flight platforms has not yet been performed. Future work will collect synchronized frame–event data during nighttime flights involving low illumination, platform vibration, rapid camera motion, attitude changes, and complex backgrounds. The reconstructed outputs will then be evaluated both for image quality and for downstream tasks such as detection, tracking, segmentation, and navigation perception. Model compression, knowledge distillation, and edge-device acceleration will also be explored to reduce model size and inference cost.

The present runtime results were obtained on an NVIDIA A100 GPU, and the model has not yet been evaluated on an embedded onboard computing platform. Consequently, practical onboard deployment will require additional validation of single-frame latency, memory use, and power consumption, together with hardware-aware compression and acceleration.

Overall, the proposed event–frame fusion reconstruction method effectively improves image quality and structural consistency in nighttime dynamic scenes. It provides a benchmark-validated approach to low-light dynamic reconstruction and a technically motivated basis for future evaluation using real nighttime UAV data.

## Figures and Tables

**Table 1:** Comparison of the existing methods on the test dataset. Bold indicates the best result. Downward and upward arrows indicate that lower and higher values are better, respectively.

Method	Performance on REDS			Performance on HQF		
	MSE ↓	PSNR ↑	SSIM ↑	MSE ↓	PSNR ↑	SSIM ↑
EDI [17]	0.182	17.037	0.664	0.336	10.906	0.515
eSL-Net [20]	0.203	15.945	0.601	0.452	7.941	0.282
E-CIR [22]	0.114	21.716	0.819	0.207	15.750	0.609
DeblurSR [23]	0.100	23.026	0.859	0.162	18.202	0.693
Ours	**0.090**	**24.079**	**0.882**	**0.138**	**19.805**	**0.748**

**Table 2:** Sensitivity analysis for the event–frame fusion weight $\lambda_{f}$ and edge-constraint weight $\lambda_{e}$. Bold indicates the best result. Downward and upward arrows indicate that lower and higher values are better, respectively.

$\lambda_{f}$	$\lambda_{e}$	REDS Validation			HQF Evaluation		
		MSE↓	PSNR↑	SSIM↑	MSE↓	PSNR↑	SSIM↑
0.3	0.01	0.096	23.790	0.873	0.148	19.430	0.736
0.3	0.05	0.094	23.890	0.876	0.144	19.580	0.741
0.3	0.10	0.097	23.730	0.871	0.150	19.350	0.734
0.5	0.01	0.092	23.950	0.879	0.141	19.660	0.744
**0.5**	**0.05**	**0.090**	**24.079**	**0.882**	**0.138**	**19.805**	**0.748**
0.5	0.10	0.093	23.910	0.878	0.143	19.600	0.742
0.8	0.01	0.099	23.610	0.868	0.153	19.200	0.729
0.8	0.05	0.096	23.780	0.873	0.149	19.390	0.735
0.8	0.10	0.100	23.550	0.866	0.155	19.120	0.727

**Table 3:** Progressive ablation of the proposed components on REDS and HQF. A checkmark indicates that the corresponding component is included. Upward arrows indicate that higher values are better. Bold indicates the best result.

Configuration	MCV	L–G	CA	SA	$L_{\mathrm{edge}}$	$L_{\mathrm{blur}}$	REDS PSNR ↑	HQF PSNR ↑
Baseline							23.026	18.202
+MCV	✓						23.214	18.548
+L–G	✓	✓					23.438	18.896
+CA	✓	✓	✓				23.612	19.132
+SA	✓	✓	✓	✓			23.748	19.326
+Edge Loss	✓	✓	✓	✓	✓		23.902	19.552
**Full Model**	✓	✓	✓	✓	✓	✓	**24.079**	**19.805**

**Table 4:** Leave-one-component-out ablation results for the full model on REDS and HQF. Bold indicates the best result. Downward and upward arrows indicate that lower and higher values are better, respectively.

Configuration	REDS			HQF		
	MSE↓	PSNR↑	SSIM↑	MSE↓	PSNR↑	SSIM↑
w/o Event–Frame Fusion	0.108	22.694	0.845	0.164	18.156	0.695
w/o Local–Global Branch	0.101	23.187	0.858	0.155	18.724	0.712
w/o Attention Module	0.096	23.562	0.868	0.148	19.116	0.725
w/o Edge Constraint	0.093	23.817	0.873	0.143	19.468	0.733
w/o Blur Constraint	0.095	23.691	0.876	0.146	19.311	0.740
**Full Model**	**0.090**	**24.079**	**0.882**	**0.138**	**19.805**	**0.748**

**Table 5:** Model complexity and runtime comparison at an input resolution of 160×160. Bold indicates the best result. Downward and upward arrows indicate that lower and higher values are better, respectively.

Method	Params (M) ↓	GFLOPs ↓	Time (ms/Sample) ↓	Equivalent FPS ↑
DeblurSR	**20.002**	**163.06**	**21.183**	**47.21**
Ours	20.017	184.96	23.756	42.09

**Table 6:** Methodological comparison with representative event-guided reconstruction approaches. Bold identifies the method proposed in this work.

Method	Input Representation	Architecture or Inference Mechanism	Supervision or Constraint	Distinctive Contribution
EDI [17]	Blurred frame and events	Analytical double-integral model	Blur-formation model and regularization	Connects blur formation and events to recover a sharp sequence
eSL-Net [20]	Low-resolution blurred frame and events	Unfolded sparse-learning network	Sparse reconstruction objectives	Joint event–frame sparse modeling for denoising and super-resolution
EventSR [21]	Pure events	Three-stage reconstruction, restoration, and super-resolution	Primarily unsupervised adversarial learning	Reconstructs and super-resolves intensity images directly from events
E-CIR [22]	Blurred frame and events	Continuous time-to-intensity representation with refinement	Reconstruction and appearance consistency	Recovers a continuous sharp-video representation
DeblurSR [23]	Blurred frame and temporally encoded events	Spiking time-to-intensity representation	Reconstruction supervision	Uses an interpretable spiking representation for motion deblurring
**Ours**	Blurred frame and 39-channel event–frame voxel	Local–global reconstruction with channel–spatial selection	Event-boundary and event-guided blur alignment	Coordinates multi-statistic representation, local–global reconstruction, and dual alignment for nighttime single-image reconstruction

## Data Availability

The data presented in this study are openly available at: https://github.com/zicicvhio/EF (accessed on 20 August 2026).

## Abbreviations

The following abbreviations are used in this manuscript:

UAV	Unmanned aerial vehicle
ROI	Region of interest
FFN	Feed-forward network
BN	Batch normalization
SOTA	State of the art

## References

[1] Lee J.H., Gwon G.H., Kim I.H., Jung H.J. (2023). A Motion Deblurring Network for Enhancing UAV Image Quality in Bridge Inspection. Drones.

[2] Feng Z., Zhang J., Ran X., Li D., Zhang C. (2025). Ghost-Unet: Multi-stage network for image deblurring via lightweight subnet learning. Measurement.

[3] Zhu J., Wang M., Zhang Z., Jia C., Geng X., Shi F. (2025). Optimization of feature association strategies in multi-target tracking based on light field images. Measurement.

[4] Liu Q., Yin L., Zhan H., Lu Y., Zhu L., Long X., Wu G. (2024). High-quality direct ghost imaging of random dynamic targets based on convolutional neural network. Opt. Laser Technol..

[5] Zhou Z., Wu Z., Boutteau R., Yang F., Demonceaux C., Ginhac D. (2022). Rgb-event fusion for moving object detection in autonomous driving. arXiv.

[6] Zashchirinskaia O.V. (2020). Patterns of interrelation between perception and understanding of images and texts with different degree of blur. Atten. Percept. Psychophys..

[7] Dargan S., Kumar M., Ayyagari M.R., Kumar G. (2020). A Survey of Deep Learning and Its Applications: A New Paradigm to Machine Learning. Arch. Comput. Methods Eng..

[8] Ouyang D., He S., Zhan J., Guo H., Huang Z., Luo M., Zhang G.L. Efficient Multi-Scale Attention Module with Cross-Spatial Learning. Proceedings of the IEEE International Conference on Acoustics, Speech, and Signal Processing.

[9] Zeng N., Wu P., Wang Z., Li H., Liu W., Liu X. (2022). A Small-Sized Object Detection Oriented Multi-Scale Feature Fusion Approach with Application to Defect Detection. IEEE Trans. Instrum. Meas..

[10] Li J., Chen X., Wang X., Bi G., Nie T., Huang L. (2025). AttFeat: Attention-based Features for Infrared and Visible Remote Sensing Image Matching. IEEE Geosci. Remote Sens. Lett..

[11] Gallego G., Delbruck T., Orchard G., Bartolozzi C., Taba B., Censi A., Leutenegger S., Davison A.J., Conradt J., Daniilidis K. (2022). Event-Based Vision: A Survey. IEEE Trans. Pattern Anal. Mach. Intell..

[12] Chakravarthi B., Verma A.A., Daniilidis K., Fermüller C., Yang Y. (2024). Recent Event Camera Innovations: A Survey. arXiv.

[13] Yang Y., Pan L., Liu L. (2023). Event Camera Data Pre-training. arXiv.

[14] Guo S., Delbruck T. (2023). Low Cost and Latency Event Camera Background Activity Denoising. IEEE Trans. Pattern Anal. Mach. Intell..

[15] Wang L., Kim T.K., Yoon K.J. (2022). Joint Framework for Single Image Reconstruction and Super-Resolution with an Event Camera. IEEE Trans. Pattern Anal. Mach. Intell..

[16] Zhang K., Ren W., Luo W., Lai W.S., Stenger B., Yang M.H., Li H. (2022). Deep Image Deblurring: A Survey. Int. J. Comput. Vis..

[17] Pan L., Scheerlinck C., Yu X., Hartley R., Liu M., Dai Y. Bringing a blurry frame alive at high frame-rate with an event camera. Proceedings of the IEEE/CVF Conference on Computer Vision and Pattern Recognition (CVPR).

[18] Rebecq H., Ranftl R., Koltun V., Scaramuzza D. Events-To-Video: Bringing Modern Computer Vision to Event Cameras. Proceedings of the 2019 IEEE/CVF Conference on Computer Vision and Pattern Recognition (CVPR).

[19] Scheerlinck C., Rebecq H., Gehrig D., Barnes N., Mahony R., Scaramuzza D. Fast Image Reconstruction with an Event Camera. Proceedings of the IEEE Workshop/Winter Conference on Applications of Computer Vision.

[20] Wang B., He J., Yu L., Xia G., Yang W. (2020). Event Enhanced High-Quality Image Recovery. Computer Vision—ECCV 2020.

[21] Wang L., Kim T., Yoon K.J. (2020). EventSR: From Asynchronous Events to Image Reconstruction, Restoration, and Super-Resolution via End-to-End Adversarial Learning. arXiv.

[22] Song C., Huang Q., Bajaj C. E-CIR: Event-Enhanced Continuous Intensity Recovery. Proceedings of the IEEE/CVF Conference on Computer Vision and Pattern Recognition (CVPR).

[23] Song C., Bajaj C., Huang Q. Deblursr: Event-based motion deblurring under the spiking representation. Proceedings of the AAAI Conference on Artificial Intelligence.

[24] Zhu L., Wang X., Chang Y., Li J., Huang T., Tian Y. Event-based video reconstruction via potential-assisted spiking neural network. Proceedings of the IEEE/CVF Conference on Computer Vision and Pattern Recognition (CVPR).

[25] Stoffregen T., Scheerlinck C., Scaramuzza D., Drummond T., Barnes N., Kleeman L., Mahony R. Reducing the sim-to-real gap for event cameras. Proceedings of the European Conference on Computer Vision.

[26] Kim T., Cho H., Yoon K.J. CMTA: Cross-Modal Temporal Alignment for Event-Guided Video Deblurring. Proceedings of the Computer Vision—ECCV 2024.

[27] Luo W., Zhang C., Yu L. Event-Based Motion Deblurring with Dual Channel Attention. Proceedings of the Computer Vision—ECCV 2024.

[28] Song G., Gai S., Da F. (2025). Memory-based gradient-guided progressive propagation network for video deblurring. Vis. Comput..

